## [Peer Review File · Nature Communications]

REVIEWER COMMENTS

Reviewer #1 (Remarks to the Author):

With the global climate changes, understanding how plants respond to elevated ambient temperatures is becoming more and more important for sustainable agriculture. Plant hormones, such as auxin, GA and BR have been reported to be involved in regulating thermoresponsive hypocotyl growth in Arabidopsis. In the manuscript by Zhu et al., the authors found that another phytohormone, JA, is also important for plant thermomorphogenesis. The well-characterized JA signaling pathway represses hypocotyl/petiole growth in Arabidopsis. Warm temperature upregulates the expression of JA catabolic enzymes, such as JOX and ST2A, therefore, tunes down the JA pathway and promotes thermoresponsive growth, which seems to be also conserved in wheat seedlings. Altogether, the genetic and metabolic profiling experiments support that JA catabolism is important for thermomorphogenesis. However, there are still some concerns to be addressed to possibly improve the paper.

The major concern is about the assays on JAZ accumulation in plant materials under different temperature conditions. Destabilization of JAZ is a key switch for JA signaling. The manuscript has used two methods to try to demonstrate the more accumulation of JAZ proteins under warm temperature conditions. However, it is very difficult to see the Jas9-VENUS signals (Fig. S11a-b) except the obvious signals of H2B-RFP. Better confocal images are needed to strengthen the conclusion. Western blotting is a good way to show the differences in protein accumulation, but quantified data from three independent blots is required for Fig. S11c. Since the transgenic plants harbor both Jas9-VENUS and H2B-RFP, antibody against RFP might be a better control than Ponceau staining. The manuscript showed the staining for 35S:JAZ1-GUS (Fig.1j), again quantitative data is missing. Because GUS is optimized for its stability to serve as a reporter for promoter activity, any fusion proteins with GUS may not reflect the stability of the fusion proteins. If the authors still wish to keep Fig. 1j, more supporting data is needed, for example, the transgene expression should be comparable among different samples.

Minor points:

- 1) Abstract Line 32, it is suggested to specify the effects, not just <affects>.
- 2) The second leaf length was not visible in Fig. 1b at 14 °C, but it is quite obvious in WT plants at 14 °C (Fig. S4c) or at 18 °C (Fig. S1d). It is important to show that no difference was observed between transgenic plants and WT plants at lower temperature. A different temperature condition higher than at 14 °C is suggested for Fig. 1b, or older plants is suggested when temperature condition is maintained at 14 °C.
- 3) More specified title is needed for the legend of Fig. 2. In Fig. 2c, the expression of AtORP3 is included, which is not mentioned in the text for in the legend.
- 4) The inducible expression of AtST2A by warm temperatures is reported in previous studies, the related reference should be cited in the text.
- 5) In Fig. S7, IAA29 is mentioned in the legend but the data is missing.
- 6) Fig. S21e, it is difficult to understand that the defects in thermoresponsive hypocotyl growth in jox-Q are weaker than that in the single mutant plants (Fig. 1h). Perhaps different phenotypic conditions were used, the hypocotyl length of WT plants is different between two figures.

Reviewer #2 (Remarks to the Author):

The authors present their study on the importance of jasmonate catabolism for warm-temperature mediated plant growth. The authors tackle this problem through a mix of molecular-genetic studies in arabidopsis, complemented with pharmaceutical and phenotypic studies in wheat.

The authors begin by showing that the second leaf of wheat undergoes temperature-dependent elongation in a manner analogous to arabidopsis hypocotyls. Both of these elongation responses are inhibited to a large extent by exogenous MeJA. The inhibition by exogenous MeJA is dependent on the canonical JA signalling pathway. Mutant arabidopsis that either produce no JA, have reduced JA signalling or overexpress JAZ3-GFP have a slightly exaggerated hypocotyl elongation at warm temperatures. At warm temperatures, there is an accumulation of 35S:JAZ1-GUS in the hypocotyl, suggesting that JA signalling is reduced in these tissues. The authors show that several JA-catabolic enzymes (JOX1-3 and ST2a) are upregulated at warm temp. Crucially, mutants that are deficient in JA catabolism show reduced hypocotyl elongation at warm temperature. The authors conclude that catabolism of JA is required for a full thermomorphogenic response.

The authors work is an important contribution to the field of plant science and would likely be eagerly read by researchers in the fields of thermomorphogenesis and plant defence signalling. The novelty of these findings is slightly diminished due to the publication of similar findings on the role of JA catabolism in shade-avoidance by Fernández-Milmanda et al (Ref 8). Fernández-Milmanda et al found that in shade, ST2a is dramatically upregulated and that it is required for shade-induced hyponasty. The upregulation of ST2a occurs is mediated by PIF transcription factors. Shade-avoidance and thermomorphogenesis share many signal components and the stabilisation of PIFs underlies the morphological effect of both cues. It is therefore not surprising that ST2a is also upregulated by warm temperatures, nor that MeJa can inhibit thermomorphogenic responses. I do not doubt that the work in the present study was developed independently of the work of Fernández-Milmanda et al and both studies help to confirm the validity of each other's results. In the present study, the complementary work in wheat does provide an important novel aspect. This is a commendable approach and really enhances the impact of this work.

Most of the results in the manuscript are of high quality, with robust statistical analysis. In my opinion there are a few points in the presentation of the results that could help to enhance readability. For example, the authors present multiple figures representing the JA metabolite profiles of three varieties of wheat and arabidopsis. This information is spread between figure 2 and supp figures 3, 13, 15, 16, 17 and 18. In its current format the reader requires an intimate knowledge of where all these metabolites fit into the JA metabolism/ catabolism pathway in order to understand the significance of each individual plot. It may help the reader if the authors provide a diagram of the JA synthesis/ catabolism pathway with the enzymes required for each step. If the JA metabolite profile for a single species is plotted around this figure, the reader can easily understand at a glance how changes in temperature change the flux in this pathway. I have attached an example figure for reference.

After plotting the results in this way, I noticed something that does raise some important questions for the interpretation of these results. The authors claim that an increase in JOX and ST2a expression at warm temp promotes JA catabolism. If this were true, we should expect an increase in sulphated JA at warm temp. While this is the case in Chinese Spring wheat, temperature has no detectable effect on 12HSO₄-JA or 12OH-JA in Col-0. This suggests that the repression of JA and JA-Ile Col plants at warm temp may occur through a different mechanism.

There are also a few places in the text that could be tightened to avoid that the data in this manuscript is not over-sold.

Line 85: "Wheat seedlings overexpressing Arabidopsis or wheat 12-OXOPHYTODIENOATE REDUCTASE 3 (OPR3), a key enzyme in JA biosynthesis, which consequently display elevated 3-oxo-2-(2-(Z)-pentenyl) cyclopentane-1-butyric acid (OPC4) levels and (partially) elevated JA levels (Supplementary Fig. 3)"

The data supporting an increase in OPC4 levels in the Tr-3 and Tr-18 lines is too variable to draw any strong conclusions and I'm not convinced there is any effect on JA levels in these lines. I would like the authors to change this sentence to reflect the uncertainty in these results.

Line 143: "To test this hypothesis, we measured JA-Ile levels in both Arabidopsis and wheat seedlings exposed to high temperature. This indeed revealed that high temperature leads to a reduction in JA-Ile levels (Fig. 2a and Supplementary Fig. 13)."

This statement is clearly holds true for arabidopsis, but again, the large variation between wheat samples make it difficult to make this claim. This sentence should also be changed to reflect the uncertainty of these result.

Minor comments:

>Why did the authors use 256 seedlings for the MeJA treated plants in Figure 1D? (as oppose to 20-30 seedlings for most other experiments)

>The sentence in line 127 is unclear. Splitting it into several parts may help readability.

>Please increase brightness of Sup Fig 11A.

> The relative increase in COI1 stability at warm temperature (S.Fig 11C) is much greater than the increase in Jas9 stability (S.Fig 12). Does this have any consequences for the interpretation of the rest of the results?

> 35s:JAZ3-deltaJas, expressing a stabilised form of JAZ3, does not have a longer hypocotyl than WT at 28°C. Do the authors have any explanation for why this might be?

Scott Hayes

Reviewer #3 (Remarks to the Author):

This article attempts to explain the natural phenomenon of elongated hypocotyl growth of plants under elevated temperature at the molecular and biochemical level. The finding adds to growing body of evidence showing involvement of the signaling components of JA and its metabolism, particularly, catabolism, in controlling plant growth responses under abiotic stress conditions. The finding is novel and authors provide large amount of data that are appropriate to understand the mechanism in detail. The paper is clearly written in a logical manner and data support their claims. I have no major objection except a few minor questions and comments. This work will be of interest to broad plant community. Nicely done.

Minor comments

Ln96 Fig. 1c-d does not show MeJA concentration dependency because it is based on presence or absence with one concentration for the presence.

Ln98 I think the author meant Suppl. Fig. 6c-d instead

Ln115 "Similar to 21 C....", the cited Suppl. Fig 8 does not support this. Hypocotyls of the listed mutants were not different from Col-0 at 21 C.

Ln174 Supplementary Fig. 19 (contrary to Fig. 2c-d) does not show that ST2A expression is upregulated by warm temperature. In the Figure legends, authors included sample collection times begin different either at dawn or during day for those two figures, perhaps implying that those provide the explanation. However, there is no explicit mention about this in the body text.

Discussion is minimal perhaps due to length limits. However, little more discussion in the light of already published works reporting related observations of growth regulation via ST2a upregulation by the phyB signaling pathway or enhanced JA-dependent insect defense responses by elevated temperature which is opposite of weakened response by increased turnover reported in this manuscript, dynamics between COI1 stabilization versus JAZ stabilization (this work) by warm temperature or link between JAZ and

phyB, etc., could put this into a broader perspective and help the reading community to understand the surrounding issues. Some of these works are mentioned and cited in the manuscript but those were brief or indirect. What might be the implication of current finding on plant fitness under warming planet?

All experiments were conducted under constitutive conditions such as constant temperatures or constant presence or absence of MeJA, which is fine. However, I wonder whether the reported conclusions will hold also true if the experiment was set up to look at the transitions from control condition to higher temperatures or addition of the hormone. Have the authors already tried this? I don't think this is required but it would be nice to have at least one supplemental data or discussion dedicated to this for readers like me.

Reviewer #1 (Remarks to the Author):

However, there are still some concerns to be addressed to possibly improve the paper.

** The major concern is about the assays on JAZ accumulation in plant materials under different temperature conditions. Destabilization of JAZ is a key switch for JA signaling. The manuscript has used two methods to try to demonstrate the more accumulation of JAZ proteins under warm temperature conditions. However, it is very difficult to see the Jas9-VENUS signals (Fig. S11a-b) except the obvious signals of H2B-RFP. Better confocal images are needed to strengthen the conclusion.*

REPLY: We have replaced the figure panels from Fig. S11a-b with a new set of higher magnification confocal images (including an inset of a representative nucleus) as a new Supplementary Figure S12a.

** Western blotting is a good way to show the differences in protein accumulation, but quantified data from three independent blots is required for Fig. S11c. Since the transgenic plants harbor both Jas9-VENUS and H2B-RFP, antibody against RFP might be a better control than Ponceau staining.*

REPLY: We have repeated this experiment using the suggested RFP reference and we quantified three independent blots per treatment (revised Supplementary Figure S12b-c). We furthermore added data from a different experimental set-up (new Supplementary Figure S12d-e) that more clearly illustrates the increased Jas9-VENUS levels at high temperature.

** The manuscript showed the staining for 35S:JAZ1-GUS (Fig.1j), again quantitative data is missing. Because GUS is optimized for its stability to serve as a reporter for promoter activity, any fusion proteins with GUS may not reflect the stability of the fusion proteins. If the authors still wish to keep Fig. 1j, more supporting data is needed, for example, the transgene expression should be comparable among different samples.*

REPLY: We have added quantitative data for GUS activity in Figure 1k. We also added JAZ1:GUS transgene expression data, which is comparable, in Supplementary Figure 11a.

Minor points:

1) Abstract Line 32, it is suggested to specify the effects, not just .

REPLY: We have added more details to the abstract.

2) The second leaf length was not visible in Fig. 1b at 14 °C, but it is quite obvious in WT plants at 14 °C (Fig. S4c) or at 18 °C (Fig. S1d). It is important to show that no difference was observed between transgenic plants and WT plants at lower temperature. A different temperature condition higher than at 14 °C is suggested for Fig. 1b, or older plants is suggested when temperature condition is maintained at 14 °C.

REPLY: We initially aimed to always have a 10 °C difference between control and treatment condition (maximal growth temperature). Unfortunately, differences probably arise, in part, because of differences in experimental set-up. For reasons that are not clear to us, our mock DMSO treatment (previously Fig. S4c) caused some differences as opposed to Fig. 1b / Fig. S1d where we used water. Nevertheless, as suggested, we have now replaced Fig. 1b including 18°C as control temperature.

3) More specified title is needed for the legend of Fig. 2. In Fig. 2c, the expression of AtORP3 is included, which is not mentioned in the text for in the legend.

REPLY: We have updated the Fig. 2 and Fig. 3 (previously combined in Fig. 2) legend titles. The expression of AtOPR3 in the old Fig.2c was moved to Supplementary Fig.14e.

4) The inducible expression of AtST2A by warm temperatures is reported in previous studies, the related reference should be cited in the text.

REPLY: We have cited the relevant reference in the text.

5) In Fig. S7, IAA29 is mentioned in the legend but the data is missing.

REPLY: We corrected the mistake in the figure legend for Supplementary Fig.7, as IAA29 data is shown in Fig. 1f.

6) Fig. S21e, it is difficult to understand that the defects in thermoresponsive hypocotyl growth in *jox-Q* are weaker than that in the single mutant plants (Fig. 1h). Perhaps different phenotypic conditions were used, the hypocotyl length of WT plants is different between two figures.

REPLY: We have now phenotyped the *jox1-4* single and *jox-Q* pentuple mutant again in a combined experiment and added new hypocotyl and petiole/petiole angle data in Fig. 3c-d and Supplementary Fig. 21b, 23b-d, respectively. In our new analyses, the *jox-Q* mutant phenotypes are not weaker than the phenotypes of single mutants, but also do not display a stronger phenotype.

Reviewer #2 (Remarks to the Author):

Most of the results in the manuscript are of high quality, with robust statistical analysis. In my opinion there are a few points in the presentation of the results that could help to enhance readability.

** For example, the authors present multiple figures representing the JA metabolite profiles of three varieties of wheat and arabidopsis. This information is spread between figure 2 and supp figures 3, 13, 15, 16, 17 and 18. In its current format the reader requires an intimate knowledge of where all these metabolites fit into the JA metabolism/ catabolism pathway in order to understand the significance of each individual plot. It may help the reader if the authors provide a diagram of the JA synthesis/ catabolism pathway with the enzymes required for each step. If the JA metabolite profile for a single species is plotted around this figure, the reader can easily understand at a glance how changes in temperature change the flux in this pathway. I have attached an example figure for reference.*

REPLY: We have taken this comment into account, and added a schematic of the JA pathway to a new Figure 2 (and to the relevant Supplemental Figures). In addition, we organized the measurements as suggested.

** After plotting the results in this way, I noticed something that does raise some important questions for the interpretation of these results. The authors claim that an increase in JOX and ST2a expression at warm temp promotes JA catabolism. If this were true, we should expect an increase in sulphated JA at warm temp. While this is the case in Chinese Spring wheat, temperature has no detectable effect on 12HSO₄-JA or 12OH-JA in Col-0. This suggests that the repression of JA and JA-Ile Col plants at warm temp may occur through a different mechanism.*

REPLY: We were indeed not able to detect 12OH-JA or a change in 12HSO₄-JA in Arabidopsis (also not at a different time of day, data not shown). Nevertheless, the *jox* and *st2a* mutants display obvious warm temperature-associated phenotypes, indicating that this pathway plays an important role. We have more explicitly stated this in the revised manuscript. In addition, we added a possible explanation for this: "This may indicate that also other regulatory mechanisms, such as glycosylation of 12OH-JA (Haroth et al, 2019, J Biol Chem 94:9858-9872), may be involved in the reduction of JA and JA-Ile levels at warm temperature in *Arabidopsis*.

There are also a few places in the text that could be tightened to avoid that the data in this manuscript is not over-sold.

** Line 85: "Wheat seedlings overexpressing Arabidopsis or wheat 12-OXOPHYTODIENOATE*

REDUCTASE 3 (OPR3), a key enzyme in JA biosynthesis, which consequently display elevated 3-oxo-2-(2-(Z)-pentenyl) cyclopentane-1-butyric acid (OPC4) levels and (partially) elevated JA levels (Supplementary Fig. 3)”. The data supporting an increase in OPC4 levels in the Tr-3 and Tr-18 lines is too variable to draw any strong conclusions and I’m not convinced there is any effect on JA levels in these lines. I would like the authors to change this sentence to reflect the uncertainty in these results.

REPLY: We agree that the levels we measured in our conditions are variable and not striking, but taking together our observed trend in combination with published data on these lines (Pigolev et al *Int. J. Mol. Sci.* 2018, 19: 3989 – Figure 2B) we are convinced that we can draw this conclusion. We nevertheless toned down this sentence as suggested: “... which display mild and variable elevated 3-oxo-2-(2-(Z)-pentenyl) cyclopentane-1-butyric acid (OPC4) and JA levels.”

** Line 143: “To test this hypothesis, we measured JA-Ile levels in both Arabidopsis and wheat seedlings exposed to high temperature. This indeed revealed that high temperature leads to a reduction in JA-Ile levels (Fig. 2a and Supplementary Fig. 13).” This statement is clearly holds true for arabidopsis, but again, the large variation between wheat samples make it difficult to make this claim. This sentence should also be changed to reflect the uncertainty of these result.*

REPLY: We have modified the sentence as suggested: “This indeed revealed that high temperature leads to a clear reduction in JA-Ile levels in *Arabidopsis* and to a reduction, although less pronounced, in JA-Ile levels in wheat”.

Minor comments:

** Why did the authors use 256 seedlings for the MeJA treated plants in Figure 1D? (as oppose to 20-30 seedlings for most other experiments)*

REPLY: This graph is a combination of multiple independent experiments that could be combined. We have now indicated this in the figure 1 legend.

** The sentence in line 127 is unclear. Splitting it into several parts may help readability.*

REPLY: We have modified this sentence.

** Please increase brightness of Sup Fig 11A.*

REPLY: We have added new confocal images in Supplementary Figure 12a.

** The relative increase in COI1 stability at warm temperature (S.Fig 11C) is much greater than*

the increase in Jas9 stability (S.Fig 12). Does this have any consequences for the interpretation of the rest of the results?

REPLY: This difference is due to the different experimental set-ups that were used. We now repeated the Jas9 stability assay using the same set-up as for COI1, and this revealed a strong stabilization of Jas9-VENUS (new Supplementary Fig. S12d-e.).

** 35s::JAZ3-deltaJas, expressing a stabilised form of JAZ3, does not have a longer hypocotyl than WT at 28°C. Do the authors have any explanation for why this might be?*

REPLY: We mainly use this line to assess insensitivity to MeJA at warm temperature. However, why this line does not have a longer hypocotyl than WT or 35S::JAZ3:GFP, we cannot explain. In the 35S::JAZ3-Δjas-GFP line or in the *jai3-1* mutant, partial sequestration of COI1 by a truncated JAZ3 prevents degradation of other JAZs after jasmonate perception (Chini et al, 2007, Nature); but, this might have additional effects that we are not aware of. However, this observation is consistent in both 35S::JAZ3-Δjas-GFP and *jai3-1* lines.

Reviewer #3 (Remarks to the Author):

I have no major objection except a few minor questions and comments. This work will be of interest to broad plant community. Nicely done.

Minor comments

** Ln96 Fig. 1c-d does not show MeJA concentration dependency because it is based on presence or absence with one concentration for the presence.*

REPLY: In this sentence, we also refer to Supplementary Fig. 6, which does show MeJA concentration dependency.

** Ln98 I think the author meant Suppl. Fig. 6c-d instead*

REPLY: We have corrected this.

** Ln115 "Similar to 21 C....", the cited Suppl. Fig 8 does not support this. Hypocotyls of the listed mutants were not different from Col-0 at 21 C.*

REPLY: We have rephrased this.

** Ln174 Supplementary Fig. 19 (contrary to Fig. 2c-d) does not show that ST2A expression is upregulated by warm temperature. In the Figure legends, authors included sample collection*

times begin different either at dawn or during day for those two figures, perhaps implying that those provide the explanation. However, there is no explicit mention about this in the body text.

REPLY: We have rephrased this as follows: “In addition, expression of *JOX* and *ST2A*, genes encoding for the enzymes involved in regulating the catabolic processes leading to 12HSO₄-JA, was significantly upregulated at warm temperature in both wheat and *Arabidopsis*, but this increase in expression is time-of-day-dependent”.

** Discussion is minimal perhaps due to length limits. However, little more discussion in the light of already published works reporting related observations of growth regulation via *ST2a* upregulation by the *phyB* signaling pathway or enhanced JA-dependent insect defense responses by elevated temperature which is opposite of weakened response by increased turnover reported in this manuscript, dynamics between *COI1* stabilization versus JAZ stabilization (this work) by warm temperature or link between JAZ and *phyB*, etc., could put this into a broader perspective and help the reading community to understand the surrounding issues. Some of these works are mentioned and cited in the manuscript but those were brief or indirect. What might be the implication of current finding on plant fitness under warming planet?*

REPLY: We have updated the discussion as suggested.

** All experiments were conducted under constitutive conditions such as constant temperatures or constant presence or absence of MeJA, which is fine. However, I wonder whether the reported conclusions will hold also true if the experiment was set up to look at the transitions from control condition to higher temperatures or addition of the hormone. Have the authors already tried this? I don't think this is required but it would be nice to have at least one supplemental data or discussion dedicated to this for readers like me.*

REPLY: We have added new data in Supplementary Fig. S7a as suggested, where we provide a short term time course to follow the dynamics in JAZ1:GUS upon exposure to high temperature. We then selected a time point where a significant change in JAZ1:GUS levels upon 28°C exposure was, namely 12h, and performed qPCR on selected genes (Supplementary Fig. S7c).

REVIEWERS' COMMENTS

Reviewer #1 (Remarks to the Author):

The manuscript has been much improved, and I have no more further questions. Regarding the references reporting the upregulation of AtST2A by warm temperatures, the paper <Cell Rep. 2018, 25(7): 1718-1728> clearly shows that AtST2A is upregulated by elevated temperatures in Arabidopsis and therefore it should be cited.

Reviewer #3 (Remarks to the Author):

I would very much like to thank the authors for their revised manuscript. It is clear that they have considered the comments of the reviewers and this has resulted in a stronger, more robust study. I'm grateful the authors for adopting my suggestions for the formatting of the JA metabolite story; in my view the new format is much easier to follow and allows the reader to easily see how flux through the pathway is altered at warm temperatures.

In my view, no more experiments need to be performed on this MS. The authors have worked extremely thoroughly to ensure a well-rounded study and so I would like to recommend it for publication.

When re-reading the MS I did notice a few minor points that I would like to bring to the author's attention.

Figure S12B. At the request of Reviewer 1, the authors have probed their blot with RFP as a loading control. The bands in this blot are however extremely faint, making it difficult to assess if the samples had equal loading. Do the authors also have the ponceau stain for this repeated blot? If they do, I would suggest that they display both loading controls.

Figure S19. The authors state in the text that "this increase in expression is time-of-day-dependent" but in the figure legend the time of day is not specified.

Figure S7c, first panel EXP8= EXPA8?

Line 147: is depending/ is dependent

Thanks again!

Scott Hayes

Reviewer #4 (Remarks to the Author):

I have read the rebuttal and the revised manuscript and confirm that the authors have answered all my questions and made sufficient revisions. Please correct 12HSO4-JA description in line 204. I have no further comment.

Reviewer #1

Regarding the references reporting the upregulation of AtST2A by warm temperatures, the paper <Cell Rep. 2018, 25(7): 1718-1728> clearly shows that AtST2A is upregulated by elevated temperatures in Arabidopsis and therefore it should be cited.

REPLY: We included this citation.

Reviewer #3

Figure S12B. At the request of Reviewer 1, the authors have probed their blot with RFP as a loading control. The bands in this blot are however extremely faint, making it difficult to assess if the samples had equal loading. Do the authors also have the ponceau stain for this repeated blot? If they do, I would suggest that they display both loading controls.

REPLY: We have added the stain free gel as a loading control.

Figure S19. The authors state in the text that "this increase in expression is time-of-day-dependent" but in the figure legend the time of day is not specified.

REPLY: We have specified this in the figure legends.

Figure S7c, first panel EXP8= EXPA8?

REPLY: We have modified the labelling.

Line 147: is depending/ is dependent

REPLY: We corrected this.

Reviewer #4

Please correct 12HSO4-JA description in line 204.

REPLY: We corrected this in line 201.