# Supplementary material for: Warm temperature triggers JOX and ST2A-mediated jasmonate catabolism to promote plant growth
Source: Nat Commun. 2021 Aug 10;12:4804. doi: 10.1038/s41467-021-24883-2 (PMC8355256; doi:10.1038/s41467-021-24883-2)
Supplement: Supplementary file 1 — Supplementary Information [file 41467_2021_24883_MOESM1_ESM.pdf]

SUPPLEMENTARY INFORMATION

Warm temperature triggers JOX and ST2A-mediated jasmonate catabolism to promote plant growth

Tingting Zhu, Cornelia Herrfurth, Mingming Xin, Tatyana Savchenko, Ivo Feussner, Alain Goossens, Ive De Smet

Supplementary Figures

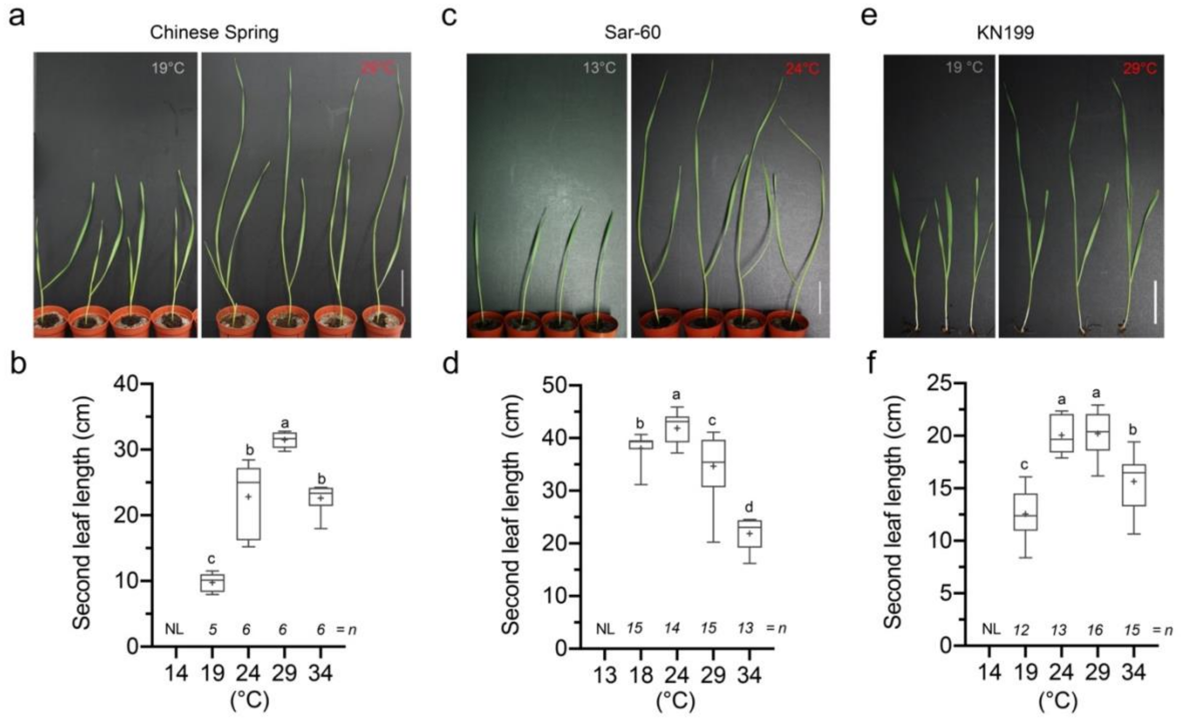

Supplementary Figure 1. Defining growth-promoting temperature in Chinese Spring (a-b), Sar-60 (c-d) and KN199 (e-f). **(a, c, e)** Representative seedlings grown at control temperature (10°C lower than temperature with maximum growth-promoting effect) and warm growth-promoting temperature (24°C or 29°C). Scale bar, 5 cm. (**b, d, f)** Second leaf length of 10-day-old Chinese Spring, Sar-60 and KN199 seedlings at the indicated temperature. Boxplots show mean as '+' and depict median with Tukey-based whiskers and outliers. NL, no visible second leaf. Different letters denote significant differences ($p < 0.05$) based on one-way ANOVA with Tukey's HSD. The exact *p*-values can be found in the Source Data. The number of individually measured seedlings (n) is indicated above the X-axis (b, d, f).

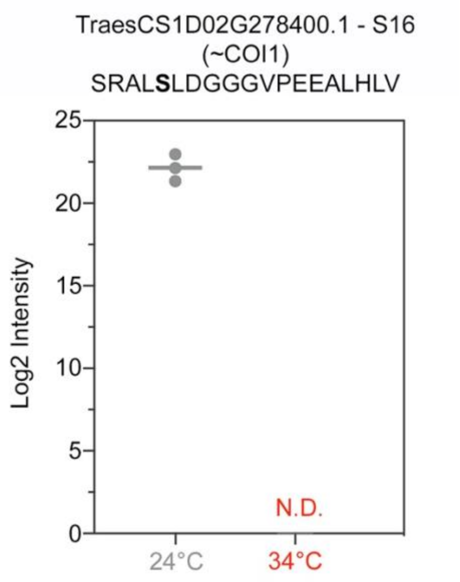

Supplementary Figure 2. Level of indicated phosphopeptide belonging to COI1 ortholog in 'Fielder' wheat seedling leaves 7 days after germination when exposed to high temperature (34°C) or control temperature (24°C) for 1 hour following growth at 24°C. Graph shows the value of the 3 individual biological replicates (dots) and the average (line). N.D., not detected. Data taken from our previously published data set[1].

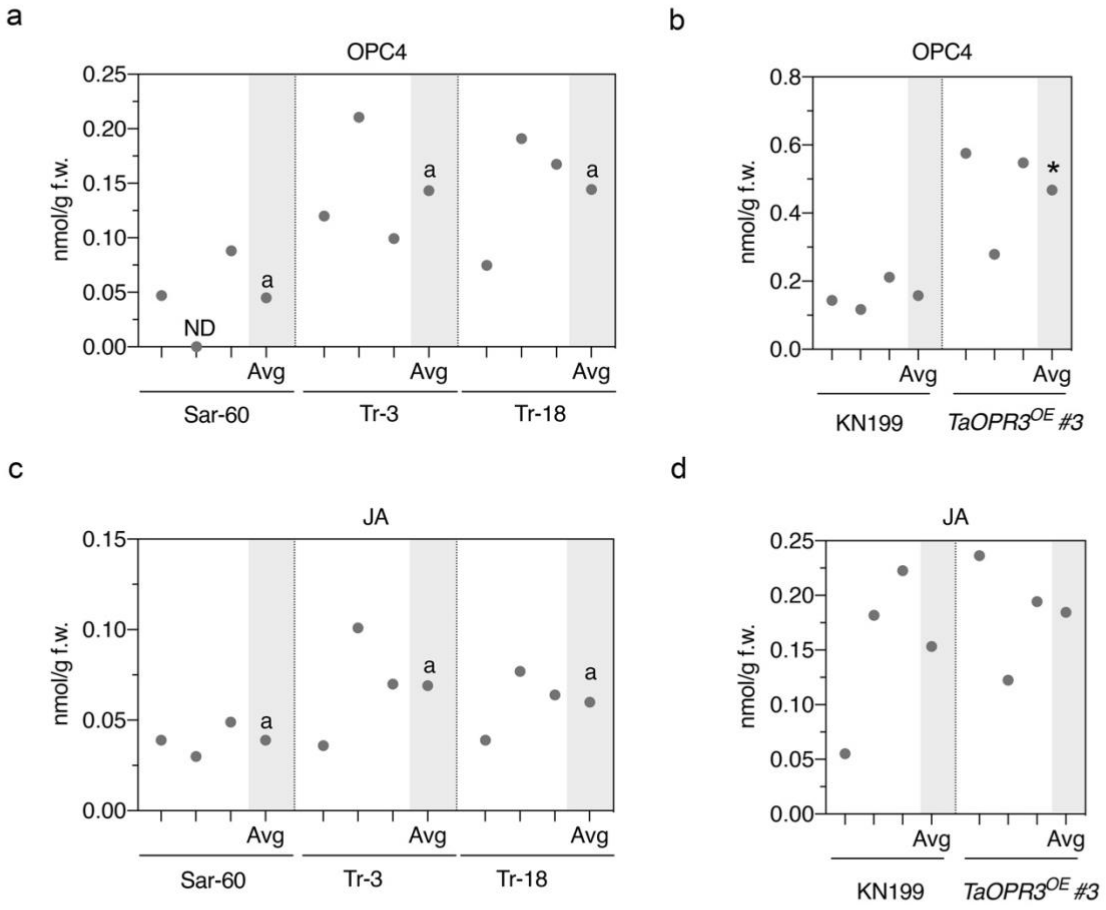

Supplementary Figure 3. (a-d) Amounts of OPC4 and JA in 10-day-old wheat seedlings at 19°C. Sar-60 (parental variety) and two *AtOPR3* overexpression lines (#Tr-3 and #Tr-18) in the Sar-60 background at 19°C (a, c). KN199 (parental variety) and a *TaOPR3* overexpression line (*TaOPR3*^{OE} #3) in the KN199 background at 19°C (b, d). Dots depict 3 biological replicates. Dot with grey background is the average (Avg) of the 3 biological replicates. ND, not detected (too low to be detected and considered as 0, and thus replaced with 0 when performing statistical analysis and calculating averages). Different letters denote significant differences ($p < 0.05$) based on one-way ANOVA with Tukey's HSD. An * denotes a significant difference ($p < 0.05$) using a Student's t-test with two-sample equal variance and one-tailed distribution. The exact *p*-values can be found in the Source Data.

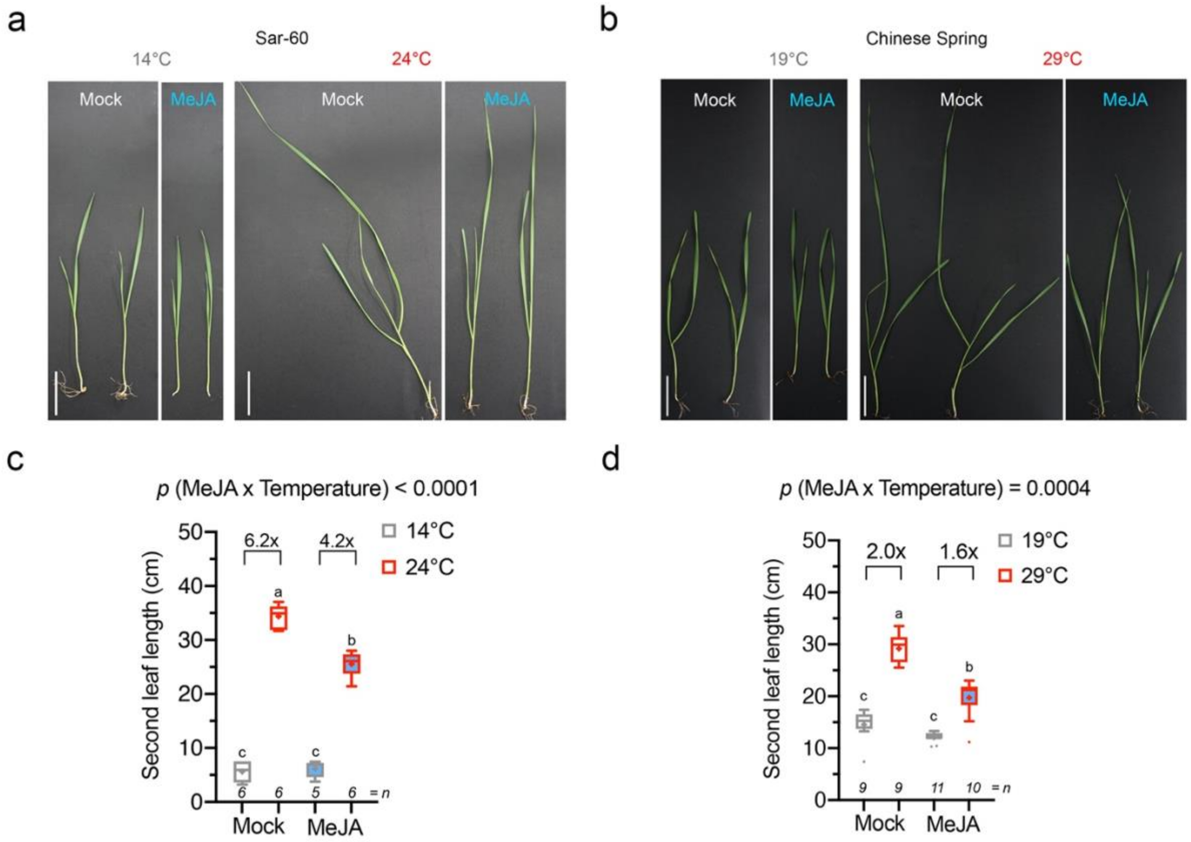

Supplementary Figure 4. (a-d) Representative images (a-b) and quantification (c-d) of the second leaf length of 10-day-old Sar-60 (a,c) and Chinese Spring seedlings (b,d) grown at control temperature (14°C or 19°C, respectively) and warm growth-promoting temperature (24°C or 29°C, respectively) in the absence or presence of 200 µM MeJA. Scale bar, 5 cm. Boxplots show mean as '+' and depict median with Tukey-based whiskers and outliers. The experiment was repeated two times with similar results. Different letters denote significant differences ($p < 0.05$) based on two-way ANOVA with Tukey's HSD with fold-change between 19°C and 29°C indicated (c, d). The number of individually measured seedlings (n) is indicated above the X-axis (c, d). The exact *p*-value (or $p<0.0001$) for the interaction (temperature × MeJA) (c, d) is shown at the top.

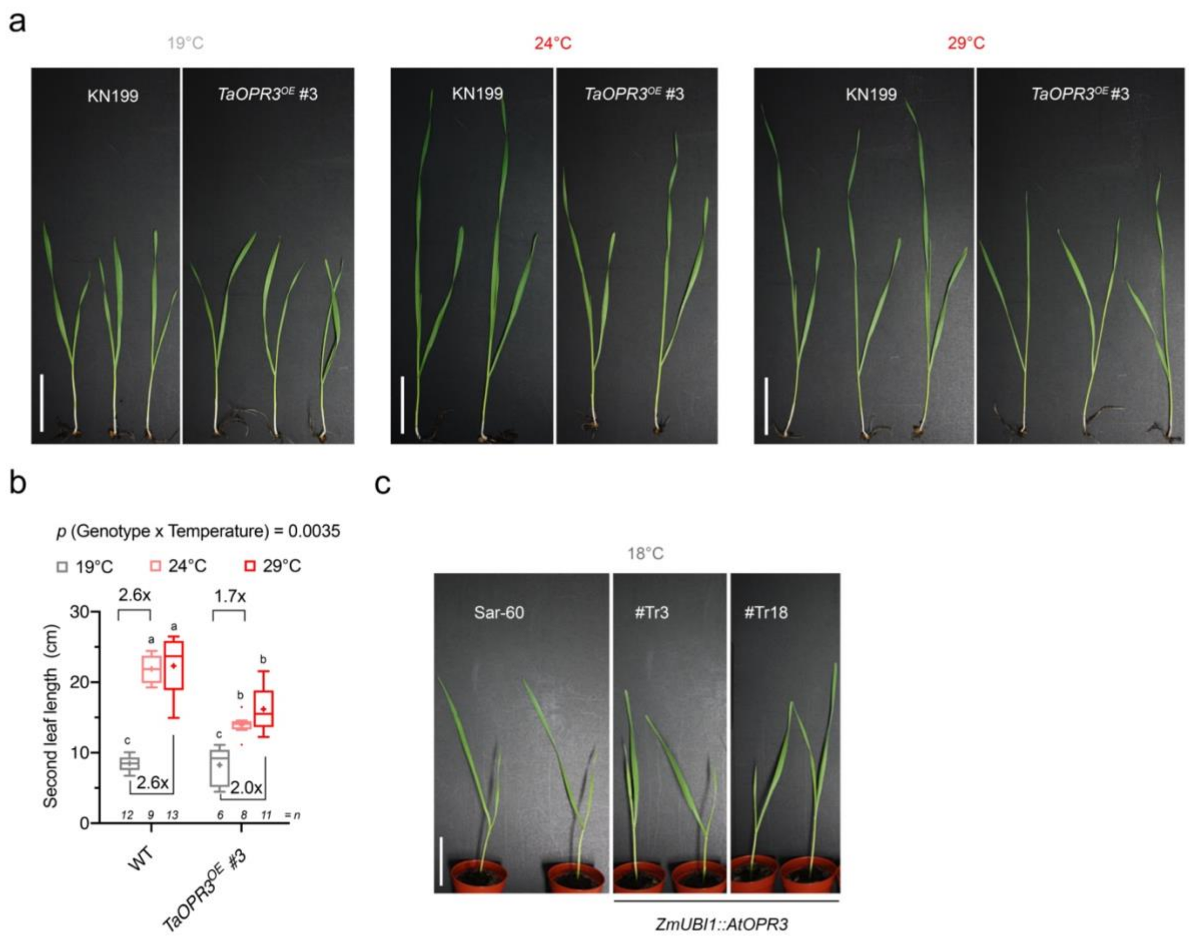

Supplementary Figure 5. (a-b) Representative images (a) and quantification of second leaf length (b) of 10-day-old KN199 (parental variety) and *TaOPR3*^{OE} (line #3) seedlings (ref. [2]) grown at control (19 °C) and warm growth-promoting temperature (24 °C or 29 °C). Scale bar, 5 cm. Boxplots show mean as '+' and depict median with Tukey-based whiskers and outliers. The experiment was repeated two times with similar results. Different letters denote significant differences ($p < 0.05$) based on two-way ANOVA with Tukey's HSD with fold-change between 19°C and 24°C or 29°C indicated (b). The number of individually measured seedlings (n) is indicated above the X-axis (b). The exact *p*-value for the interaction (genotype × temperature) (b) is shown at the top. **(c)** Representative images of 10-day-old Sar-60 (wild type) and two *AtOPR3* overexpression lines (Tr-3 and Tr-18) in the Sar-60 background at 18°C.

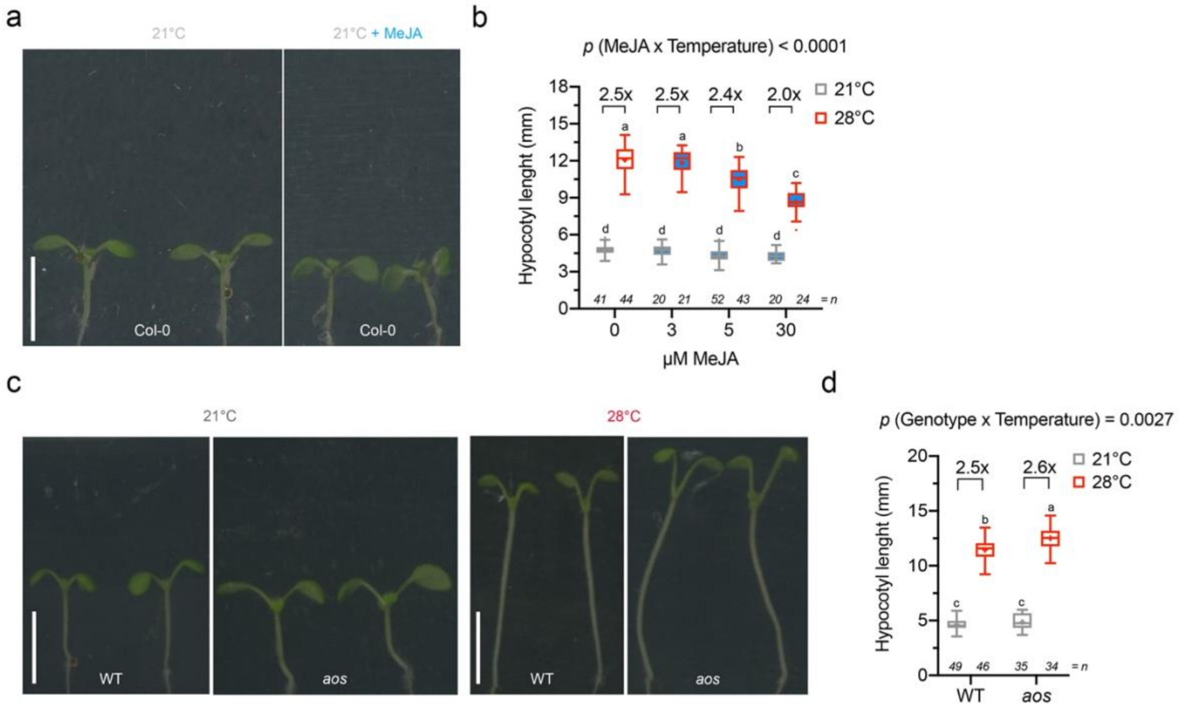

Supplementary Figure 6. (a) Representative images of 7-day-old Col-0 wild type at 21°C in the absence or presence of 30 µM MeJA. **(b)** Hypocotyl length of 7-day-old Col-0 wild type at 21°C and 28°C in the presence of different MeJA concentrations. Boxplots show median with Tukey-based whiskers and outliers. Different letters denote significant differences ($p < 0.05$) based on two-way ANOVA with Tukey's HSD and fold-change between 21°C and 28°C indicated. **(c-d)** Representative images (c) and hypocotyl length (d) of 7-day-old *aos* mutant seedlings at 21°C and 28°C. Scale bar, 5 mm. Boxplots show mean as '+' and show median with Tukey-based whiskers and outliers. Different letters denote significant differences ($p < 0.05$) based on two-way ANOVA with Tukey's HSD. The exact *p*-value (or $p < 0.0001$) for the interaction (temperature × MeJA) (b) or (genotype × temperature) (d) is shown at the top. The fold change is indicated. The number of individually measured seedlings (n) is indicated above the X-axis (b, d).

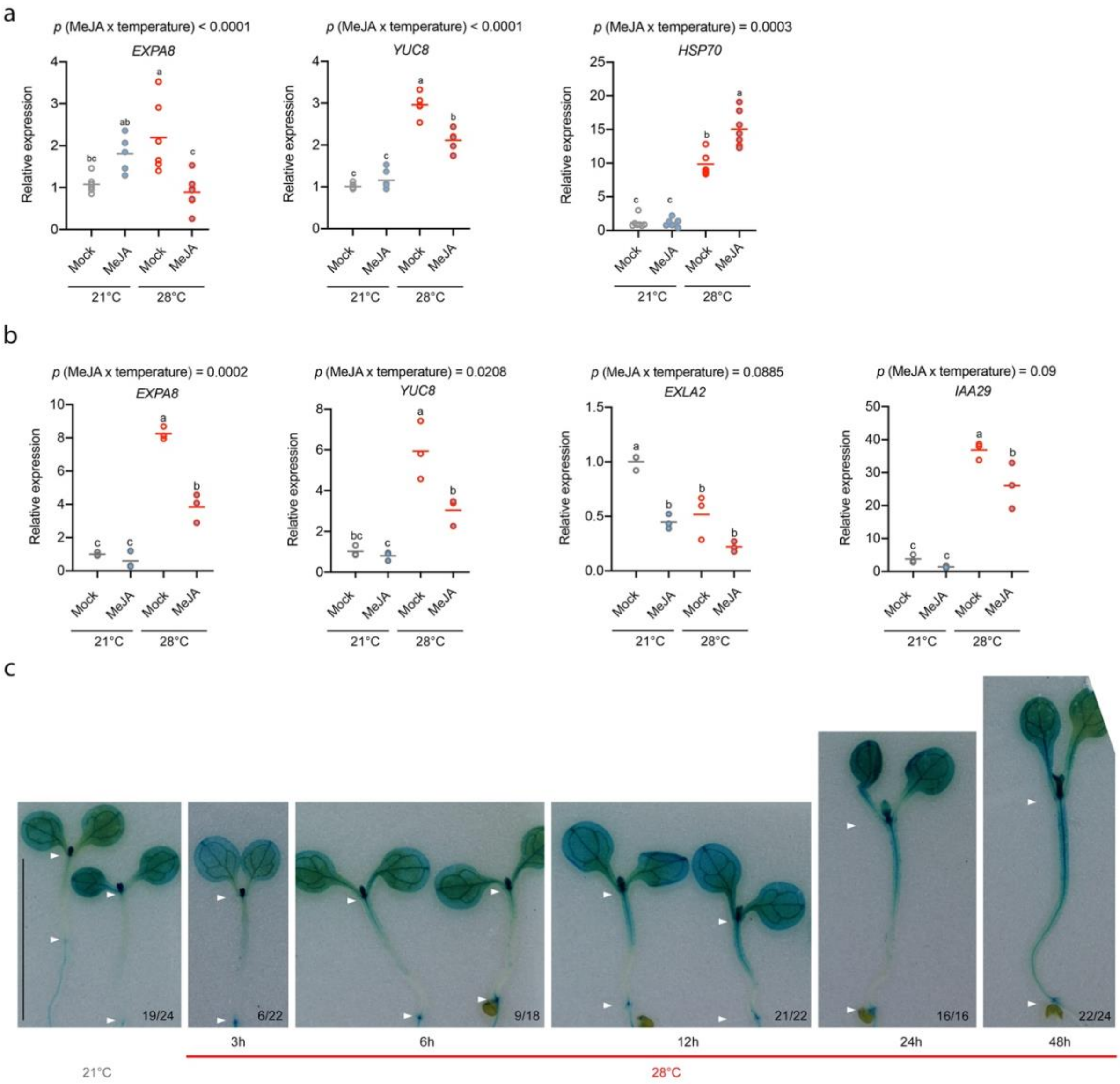

Supplementary Figure 7. (a-b) Relative expression of *EXPA8*, *YUC8* and *HSP70* in 7-day-old Col-0 wild type seedlings grown at 21°C and 28°C (a) or 5-day-old Col-0 seedlings grown at 21°C and transferred to 28°C for 12 h (b), in the absence or presence of 30 µM MeJA. Graph shows the value of the 3-6 individual biological replicates (dots) and the average (line). Different letters denote significant differences ($p < 0.05$) based on two-way ANOVA with Tukey's HSD. The exact p-value (or $p < 0.0001$) for the interaction (MeJA × temperature) is shown at the top. **(c)** Representative images of progressive GUS signal in the hypocotyl (indicated by white arrowheads) of 5-day-old *35S::JAZ1:GUS* seedlings grown at 21°C and transferred to 28°C for 0h, 3h, 6h, 12h, 24h and 48h. Scale bar, 5 mm. The numbers indicate the number of seedlings corresponding to the representative images out of total number of seedlings used for staining per condition.

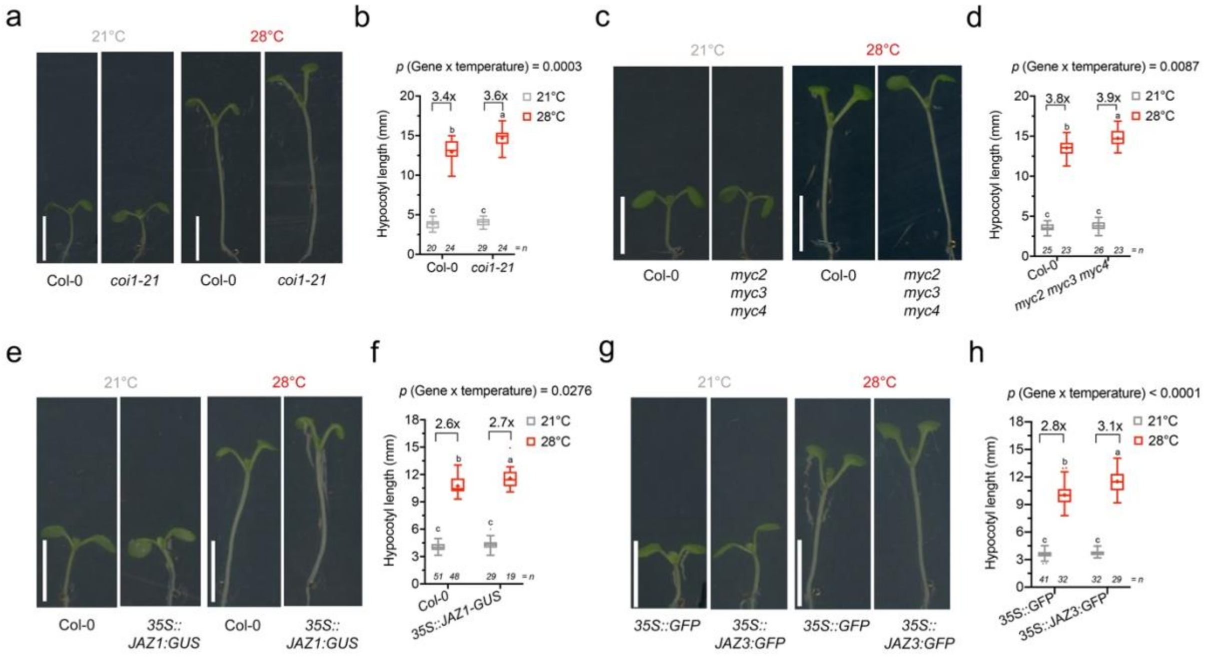

Supplementary Figure 8. (a-h) Representative pictures (a, c, e, g) and quantification of hypocotyl length (b, d, f, h) of 7-day-old *coi1-21* (a, b), *myc2 myc3 myc4* (c, d), a *35S::JAZ1:GUS* line (e, f), and a *35S::JAZ3-GFP* line (g, h) at 21°C and 28°C. Scale bar, 5 mm. All boxplots show mean as '+' and show median with Tukey-based whiskers and outliers. Different letters denote significant differences ($p < 0.05$) based on two-way ANOVA with Tukey's HSD with fold-change between 21°C and 28°C being presented (b, d, f, h). The exact p-value (or $p < 0.0001$) for the interaction (genotype × temperature) (b, d, f, h) is shown at the top. The experiment was repeated at least two times with similar results. The number of individually measured seedlings (n) is indicated above the X-axis (b, d, f, h).

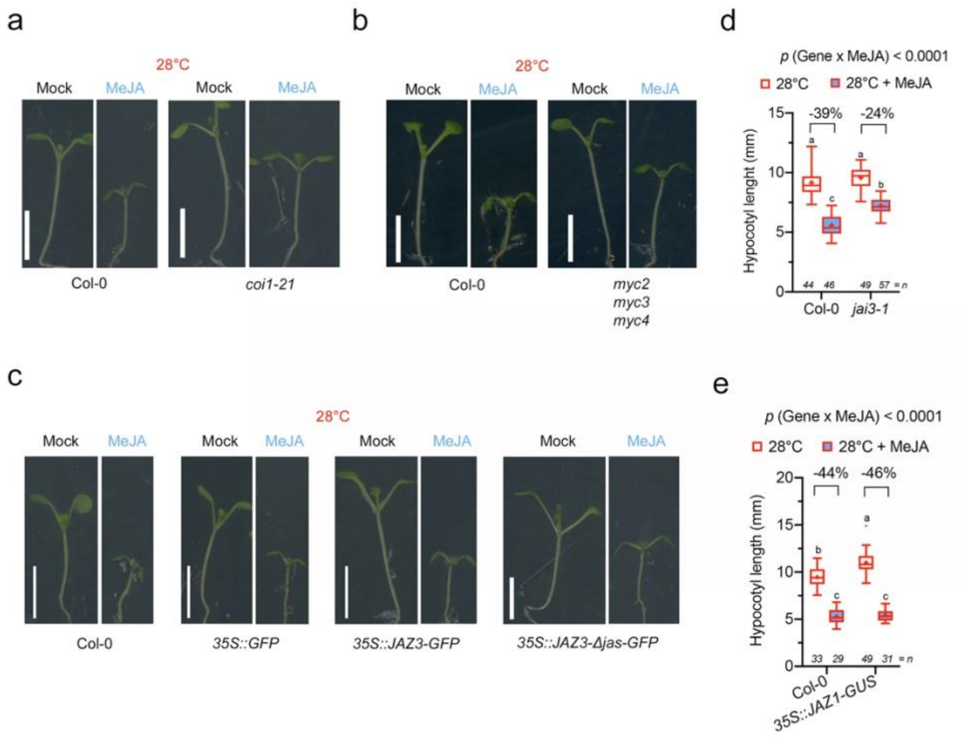

Supplementary Figure 9. (a-c) Representative pictures of 7-day-old Col-0 wild type compared to *coi1-21 (a)*, *myc2 myc3 myc4* (b), and *35S::JAZ3:GFP* or *35S::JAZ3-Δjas-GFP* (c) lines at 28°C in the absence or presence of 30 µM MeJA. Scale bar, 5 mm. **(d-e)** Quantification of hypocotyl length of 7-day-old *jai3-1* (d) and *35S::JAZ1-GUS* (e) at 28°C in the absence or presence of 30 µM MeJA. Boxplots show mean as '+' and depict median with Tukey-based whiskers and outliers. Different letters denote significant differences ($p < 0.05$) based on two-way ANOVA with Tukey's HSD. The % decrease is indicated. The number of individually measured seedlings (n) is indicated above the X-axis (d, e). The *p*-value for the interaction (genotype × MeJA) (d, e) is shown at the top.

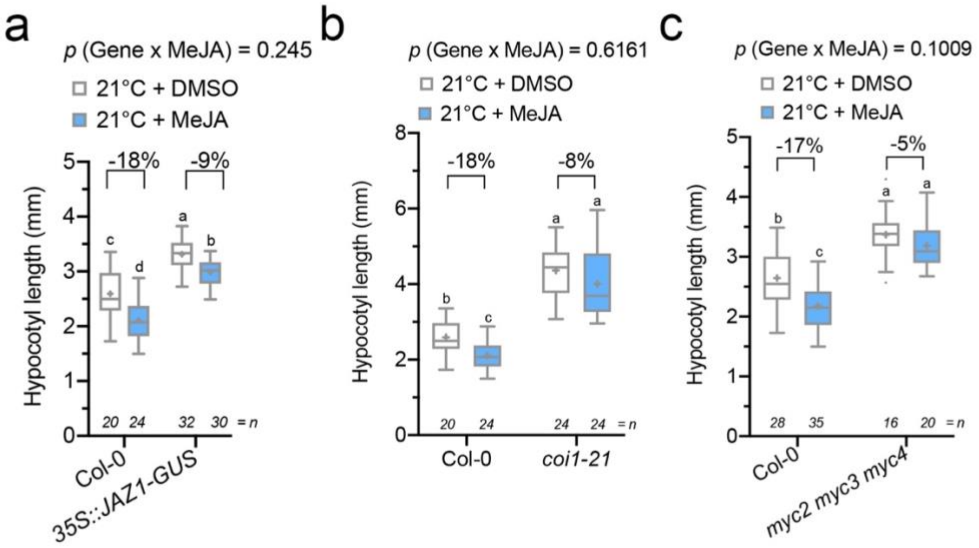

Supplementary Figure 10. (a-c) Hypocotyl length of MeJA-treated lines at 21°C. Quantification of hypocotyl length at 21°C and 21°C in presence of 30 µM MeJA for *35S::JAZ1:GUS* (a), *coi1-21* (b) and *myc2 myc3 myc4* (c) compared to the Col-0 wild type. All boxplots show mean as '+' and show median with Tukey-based whiskers and outliers. Different letters denote significant differences ($p < 0.05$) based on two-way ANOVA with Tukey's HSD. The exact *p*-value for the interaction (genotype × MeJA) is shown at the top. The % decrease is indicated. The number of individually measured seedlings (n) is indicated above the X-axis.

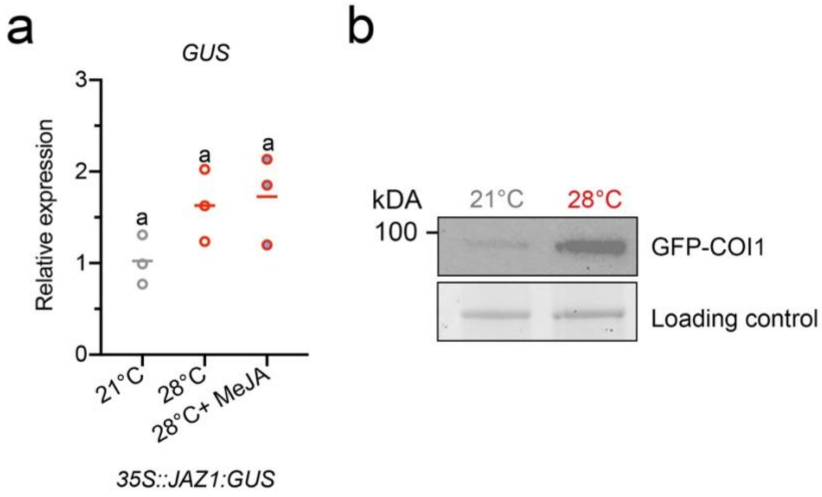

Supplementary Figure 11. (a) Relative expression of *JAZ1:GUS* (using *GUS* primers) in 5-day-old *35S::JAZ1:GUS* seedlings grown at 21°C and 28°C in the absence or presence of 30 µM MeJA. Graph shows the value of the 3 individual biological replicates (dots) and the average (line). Different letters denote significant differences ($p < 0.05$) based on one-way ANOVA with Tukey's HSD. The exact *p*-values can be found in the Source Data. **(b)** Western blot showing the analysis of total protein extracts of 7-day-old *35S::GFP-COI1* seedlings at 21°C and 28°C probed with an anti-GFP antibody. Stain-free blot image as loading control. The experiment was repeated two times with similar results.

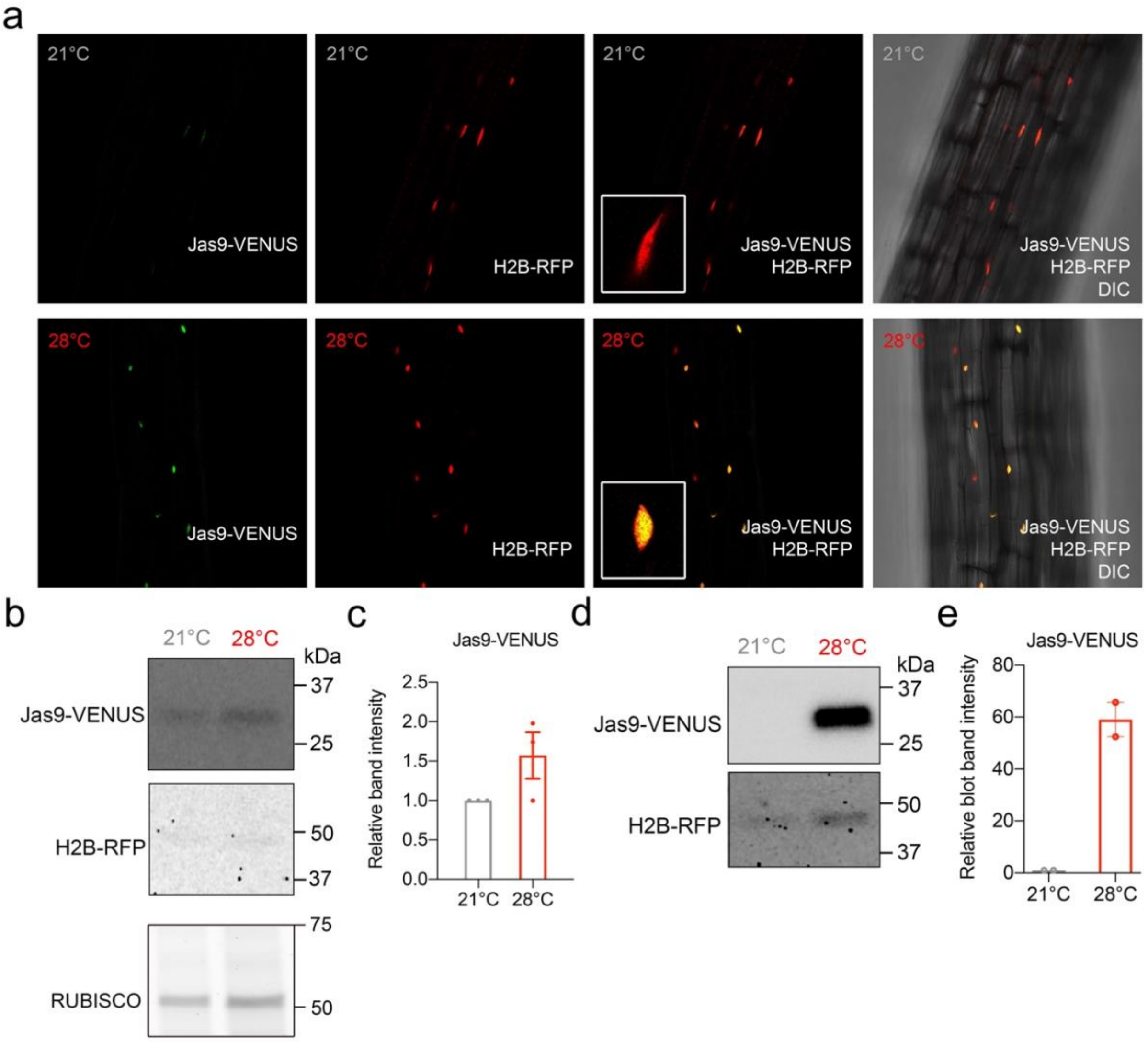

Supplementary Figure 12. (a) From left to right, Jas9-VENUS (green), H2B-RFP (red), overlay (yellow) of Jas9-N7-VENUS and H2B-RFP, and overlay of Jas9-VENUS and H2B-RFP with a DIC image of the hypocotyl cells at 21°C and 28°C. Insets show representative nucleus. DIC, differential interference contrast. **(b-c)** Western blot showing Jas9-VENUS levels in *35S::Jas9-VENUS* seedlings treated for 20 hours at 21°C and 28°C and probed with an anti-GFP antibody and H2B-RFP levels as loading control. Stain-free gel highlighting RUBISCO is also shown. (b). Bar diagram of protein quantification (with H2B-RFP as reference) shows three independent biological replicates (individual dots) with standard error of the mean (c). **(d-e)** Western blot showing Jas9-VENUS levels in *35S::Jas9-VENUS* seedlings treated for 6 hours at 21°C and 28°C and probed with an anti-GFP antibody and H2B-RFP levels as loading control (d). Bar diagram of protein quantification shows mean of two independent biological replicates (individual dots).

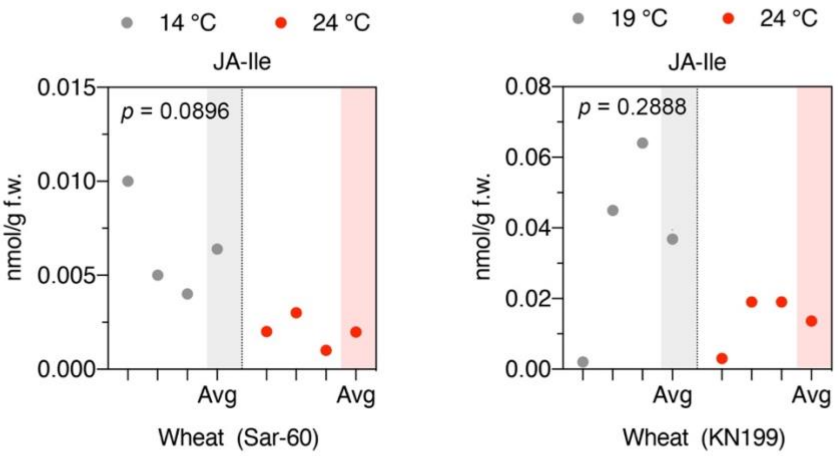

Supplementary Figure 13. Amounts of JA-Ile in 10-day-old Sar-60 and KN199 shoots at control temperature (14°C or 19°C) and warm growth-promoting temperature (24°C). Samples were collected during the day. Dots depict 3 biological replicates and the average (Avg) as the dot with grey or red background. ND, not detected (too low to be detected and considered as 0, and thus replaced with 0 when performing statistical analysis and calculating average). No significant differences ($p < 0.05$) using a Student's t-test with two-sample equal variance and one-tailed distribution. The exact *p*-value is shown in the graph.

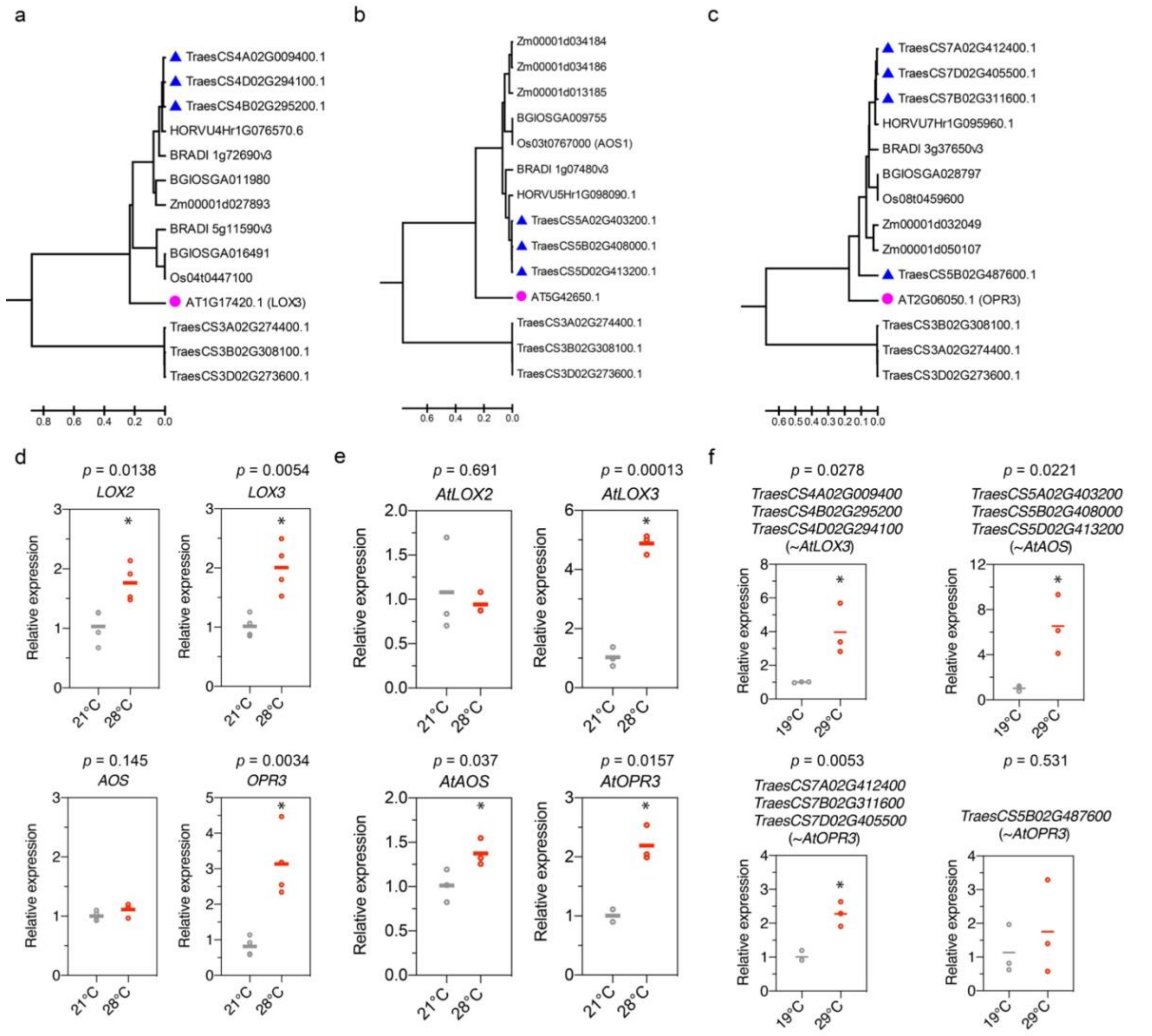

Supplementary Figure 14. (a-c) Phylogenetic trees for LOX3 (a), AOS (b) and OPR3 (c) orthologs in *Arabidopsis*, wheat, rice, maize, *Brachypodium* and barley based on amino acid sequence. Scale represents the branch length. Some sequences are marked with a pink dot (*Arabidopsis* protein) or blue triangle (selected for qPCR). Wheat orthologs of *Arabidopsis* HSP93 (AT3G48870) are the outgroup. **(d-e)** Relative expression of JA biosynthetic genes in 7-day-old Col-0 wild type shoots at 21°C and 28°C. Samples were collected during the day (at 2.00 pm) (d) or at dawn (e). **(f)** Relative expression of genes encoding for putative wheat *LOX3*, *AOS*, *OPR3* orthologs in 10-day-old Chinese Spring shoots at 19°C and 29°C. Samples were collected during the day. Graphs in d-f depict average (line) of 3 biological replicates (dots). An * denotes significant differences ($p < 0.05$) using a Student's t-test with two-sample equal variance and one-tailed distribution. The exact *p*-value (or $p < 0.0001$) is shown at the top.

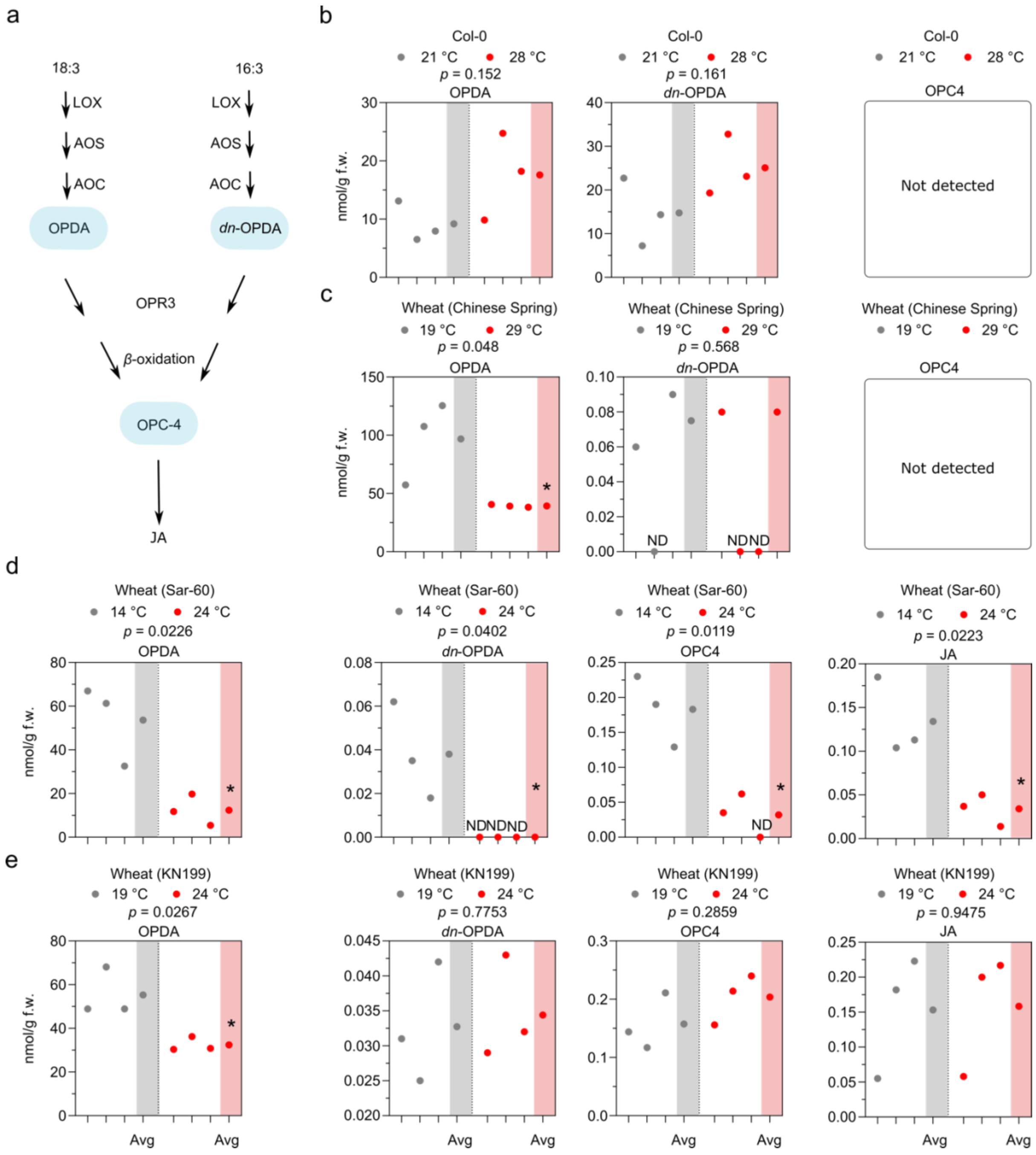

Supplementary Figure 15. (a) Schematic of JA biosynthesis pathway. **(b-e)** Amounts of OPDA, *dn*-OPDA, OPC4 and/or JA in (a) 7-day-old Col-0 wild type shoots at 21°C and 28°C and (b-d) 10-day-old Chinese Spring (b), Sar-60 (c) and KN199 (d) shoots at control temperature and warm growth-promoting temperature. Dots show 3 biological replicates and the average (Avg) with grey or red background. ND, not detected (too low to be detected and considered as 0, and thus replaced with 0 when performing statistical analysis and calculating average). An * denotes a significant difference ($p < 0.05$) using a Student's t-test with two-sample equal variance and one-tailed distribution. The exact *p*-value is shown at the top.

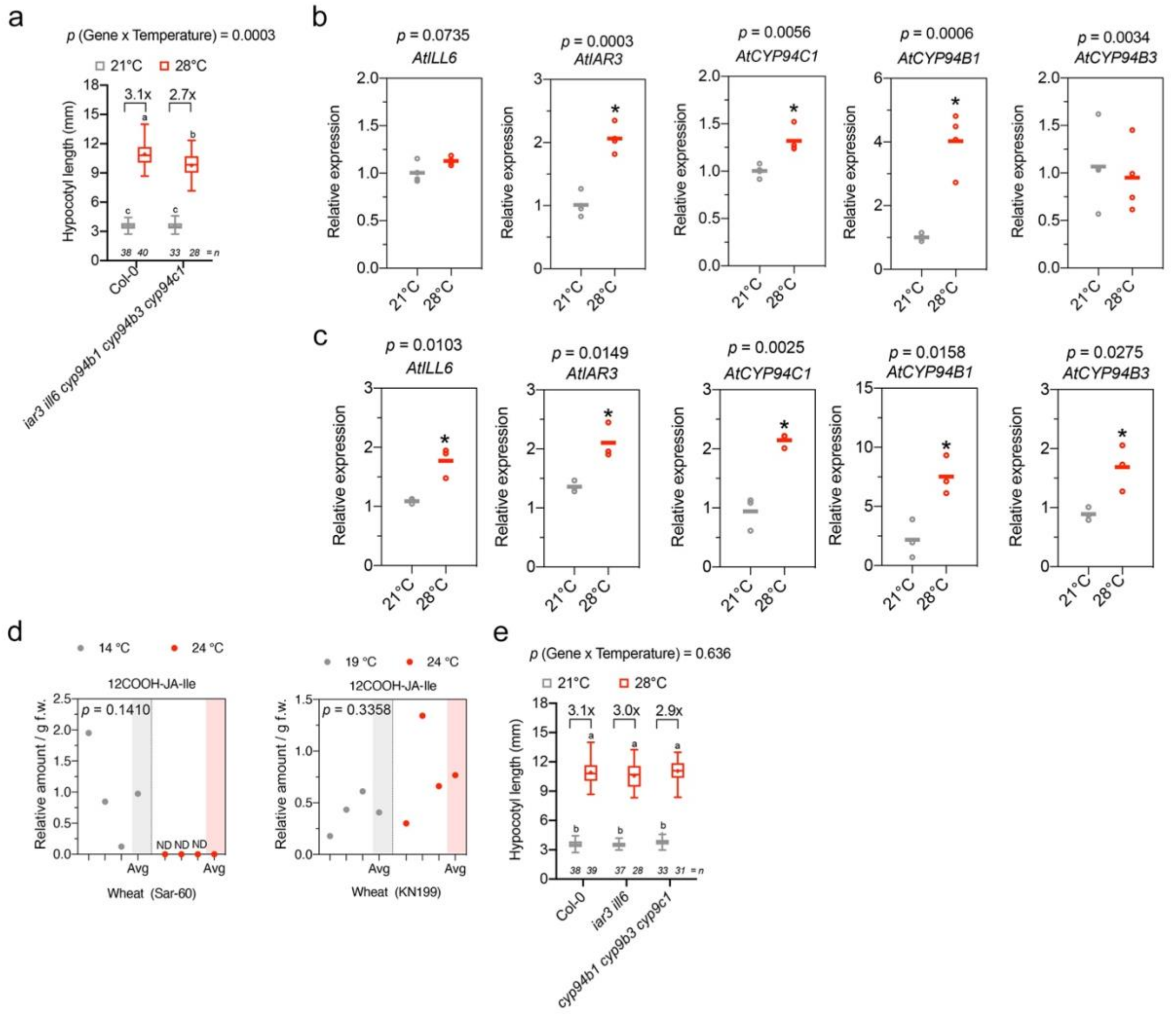

Supplementary Figure 16. (a) Hypocotyl length of 7-day-old *iar3 ill6 cyp94b1 cyp94b3 cyp94c1* pentuple mutant lines at 21°C and 28°C. Boxplots show mean as '+' and show median with Tukey-based whiskers and outliers. Different letters denote significant differences ($p < 0.05$) based on two-way ANOVA with Tukey's HSD. The exact *p*-value for the interaction (genotype × temperature) is shown at the top. The fold change is indicated (28°C versus 21°C). The number of individually measured seedlings (n) is indicated above the X-axis. **(b-c)** Relative expression of *ILL6, IAR3, CYP94C1*, *CYP94B1* and *CYP94B3* in 7-day-old Col-0 wild type shoots at 21°C and 28°C. Samples were collected during the day (at 2.00 pm) (b) or at dawn (c). Graphs depict average (line) of 3 biological replicates (dots). An * denotes a significant difference ($p < 0.05$) using a Student's t-test with two-sample equal variance and one-tailed distribution. The exact *p*-value is shown at the top or in the graph. **(d)** Relative amounts of 12COOH-JA-Ile in 10-day-old wheat shoots (Sar-60 and KN199) at control temperature and warm growth-promoting temperature. Dots depict 3 biological replicates and the average (Avg) as the dot with grey or red background. ND, not detected (too low to be

detected and considered as 0, and thus replaced with 0 when performing statistical analysis and calculating average). No significant differences ($p < 0.05$) using a Student's t-test with two-sample equal variance and one-tailed distribution. The exact *p*-value is shown in the graph. **(e)** Hypocotyl length of 7-day-old *iar3 ill6* and *cyp94b1 cyp94b3 cyp94c1* mutant seedlings at 21°C and 28°C (b). Boxplots show mean as '+' and show median with Tukey-based whiskers and outliers. Different letters denote significant differences ($p < 0.05$) based on two-way ANOVA with Tukey's HSD. The exact *p*-value for the interaction (genotype × temperature) is shown at the top. The fold change is indicated (28°C versus 21°C). The number of individually measured seedlings (n) is indicated above the X-axis.

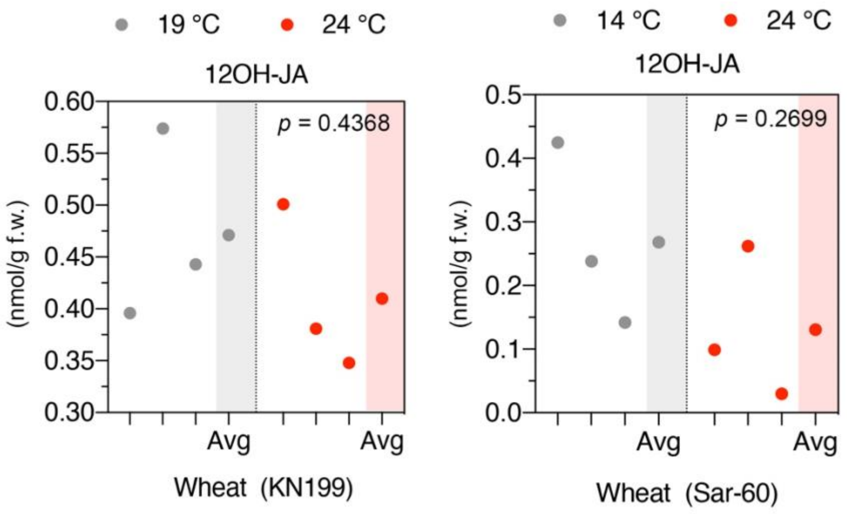

Supplementary Figure 17. Amounts of 12OH-JA in 10-day-old KN199 and Sar-60 shoots at control temperature (19°C or 14°C) and warm growth-promoting temperature (24°C). Samples were harvested during the day. Dots depict 3 biological replicates and the average (Avg) as the dot with grey or red background. No significant differences ($p < 0.05$) using a Student's t-test with two-sample equal variance and one-tailed distribution. The exact *p*-value is shown in the graph.

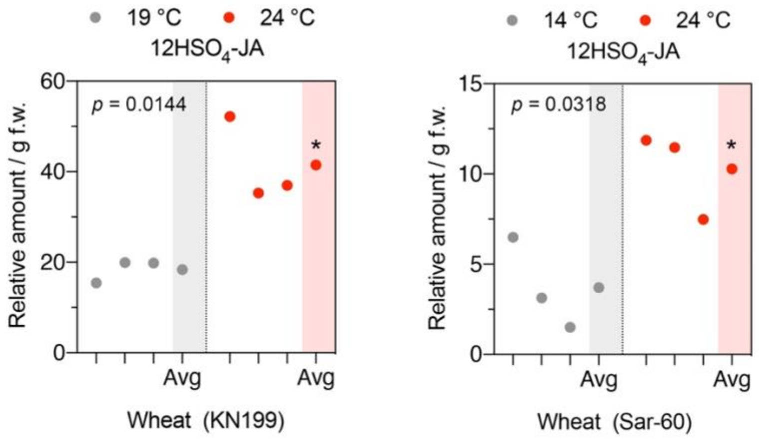

Supplementary Figure 18. Amounts of 12HSO4-JA in 10-day-old KN199 and Sar-60 shoots at control temperature (19°C or 14°C) and warm growth-promoting temperature (24°C). Dots depict 3 biological replicates and the average (Avg) as the dot with grey or red background. An * denotes a significant difference ($p < 0.05$) using a Student's t-test with two-sample equal variance and one-tailed distribution. The exact *p*-value is shown in the graph.

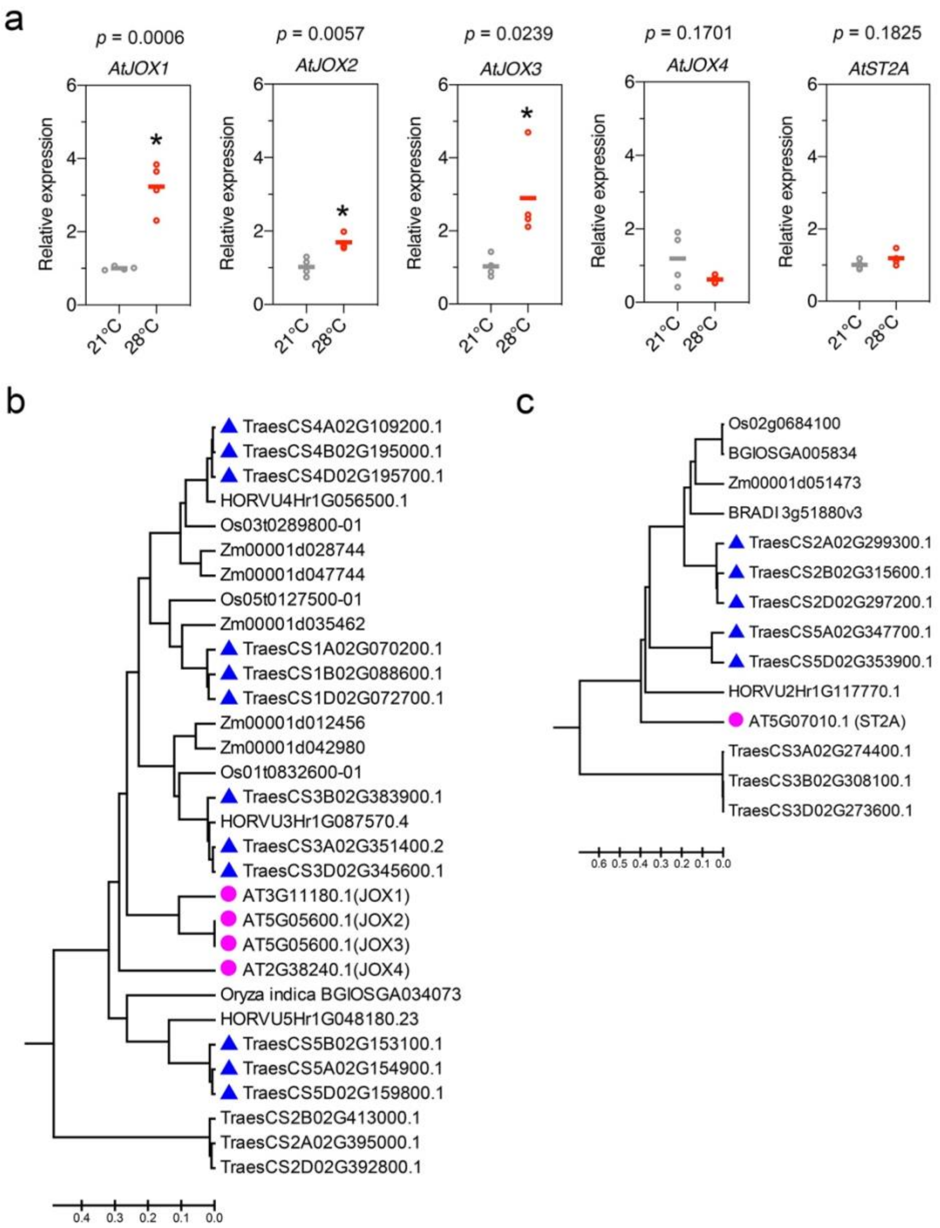

Supplementary Figure 19. (a) Relative expression of *JOX* and *ST2A* genes in 7-day-old Col-0 wild type shoots at 21°C and 28°C. Samples were collected during the day (at 2.00 pm). Graphs depict average (line) of 3 biological replicates (dots). An * denotes a significant difference ($p < 0.05$) using a Student's t-test with two-sample equal variance and one-tailed distribution. The exact *p*-value is shown at the top. **(b-c)** Phylogenetic tree for JOX (a) and ST2A (b) orthologs in *Arabidopsis*, wheat, rice, maize, *Brachypodium* and barley based on amino acid sequence. Scale rule represents the branch length. Some sequences are marked with a pink dot (*Arabidopsis* protein) or blue triangle (selected for qPCR).

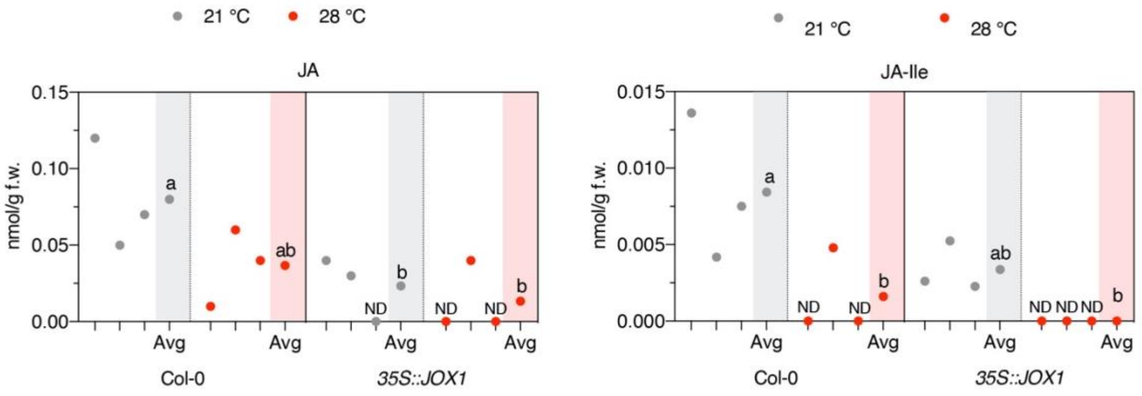

Supplementary Figure 20. Amounts of JA and JA-Ile in 7-day-old Col-0 wild type and *35S::JOX1* shoots at 21°C and 28°C. ND, not detected (too low to be detected and considered as 0, and thus replaced with 0 when performing statistical analysis and calculating average). Dots depict 3 biological replicates and the average (Avg) as the dot with grey or red background. Different letters denote significant differences (p < 0.05) based on two-way ANOVA with Tukey's HSD. The exact p-values can be found in the Source Data.

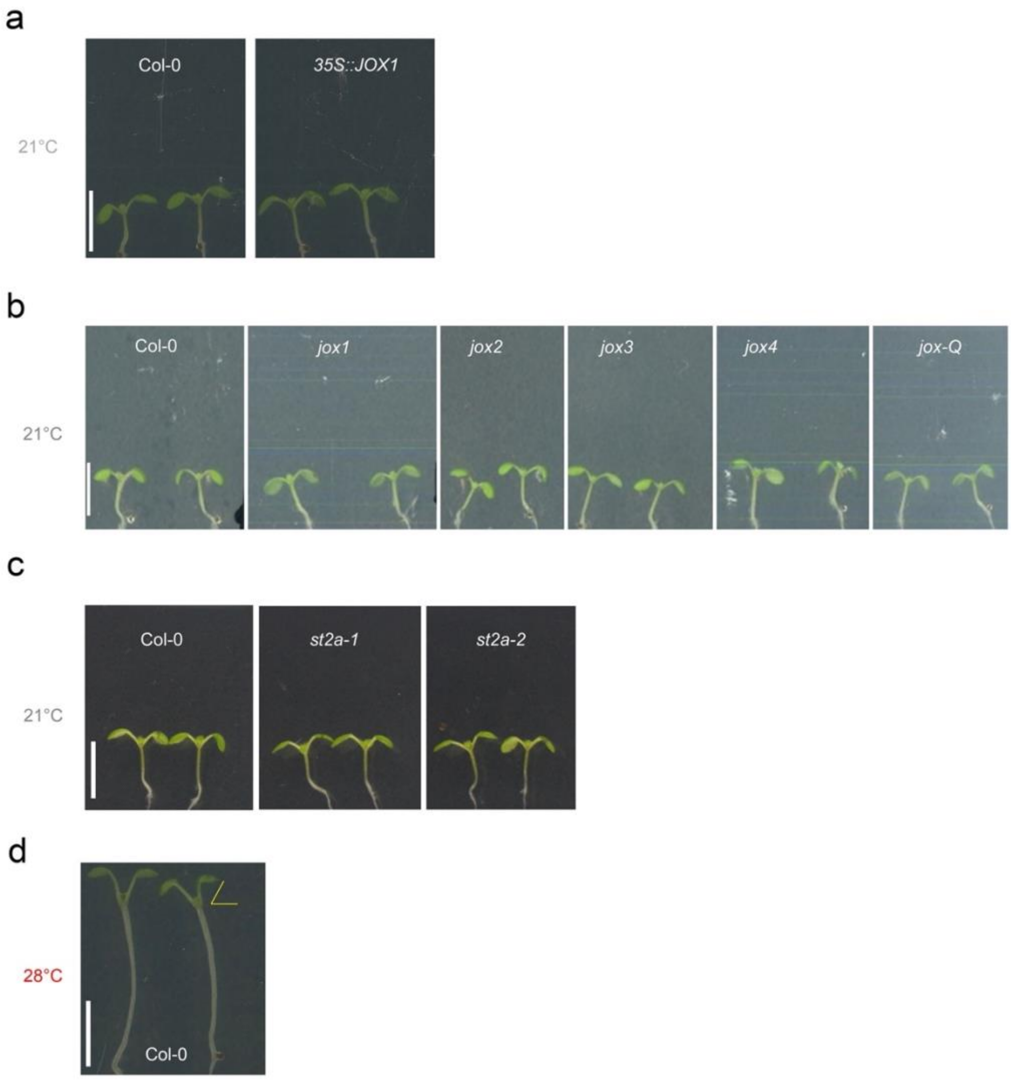

Supplementary Figure 21. (a-c) Representative pictures of 7-day-old Col-0 wild type and *35S::JOX1* (a), *jox1-4* single and *jox*-Q quadruple mutant (b), and *st2a* mutant lines (c) at 21°C. Scale bar, 5 mm. **(d)** Representative Col-0 with yellow angle indicating how petiole angle was measured. Scale bar, 5 mm.

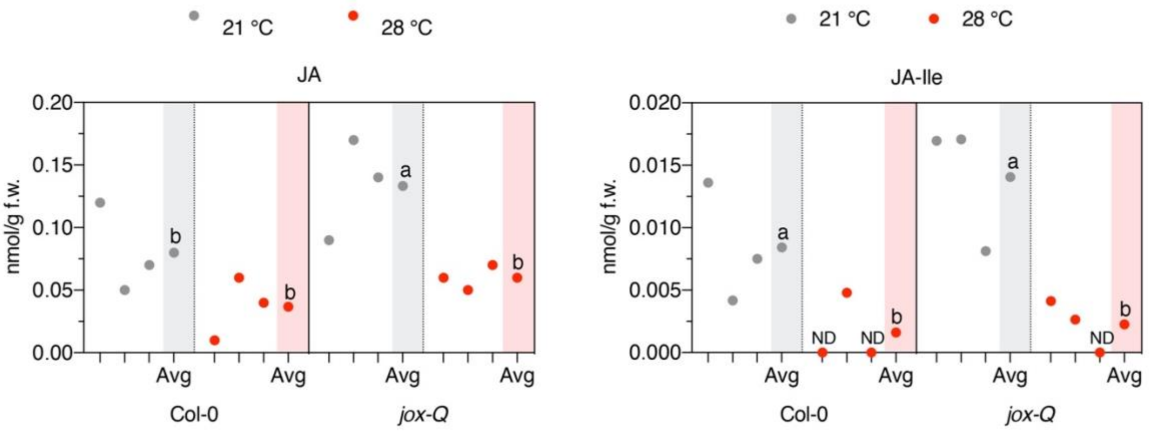

Supplementary Figure 22. Amounts of JA and JA-Ile in Col-0 wild type and *jox-Q* mutant shoots at 21°C and 28°C. ND, not detected (too low to be detected and considered as 0, and thus replaced with 0 when performing statistical analysis and calculating average). Dots depict 3 biological replicates and the average (Avg) as the dot with grey or red background. Different letters denote significant differences ($p < 0.05$) based on two-way ANOVA with Tukey's HSD. The exact *p*-values can be found in the Source Data.

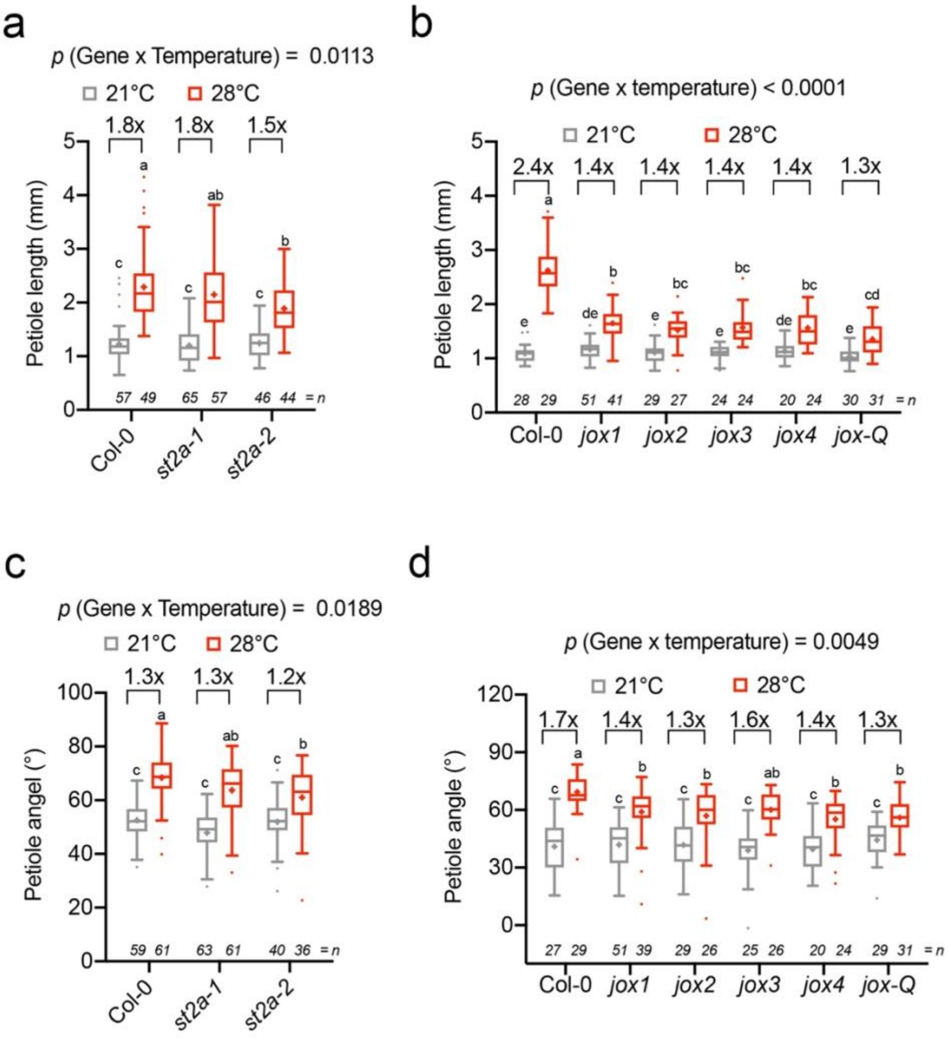

Supplementary Figure 23. (a-b) Petiole length and **(c-d)** petiole angle of 7-day-old Col-0 and *jox1-4* single and *jox*-Q quadruple (b, d), and *st2a* (a, c) mutant lines at 21°C and 28°C. All boxplots show mean as '+' and show median with Tukey-based whiskers and outliers. Different letters denote significant differences ($p < 0.05$) based on two-way ANOVA with Tukey's HSD. The fold change is indicated. The number of individually measured seedlings (n) is indicated above the X-axis. The exact *p*-value or ($p < 0.0001$) for the interaction (genotype × temperature) is shown at the top.

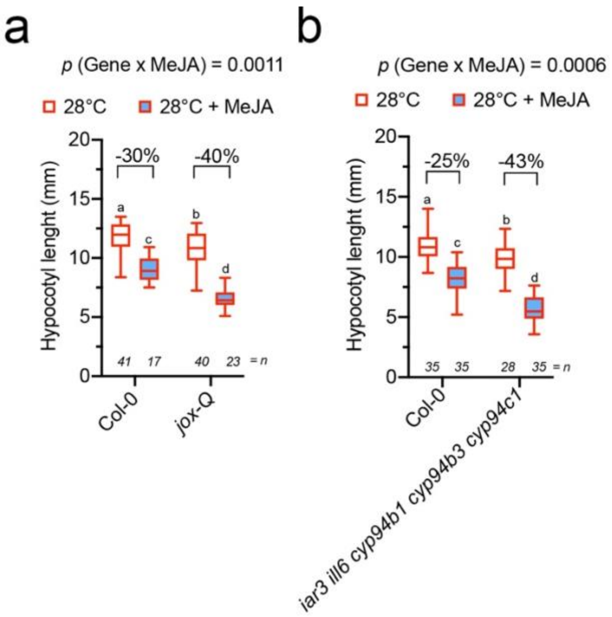

Supplementary Figure 24. Quantification of hypocotyl length of 7-day-old *jox-Q* quadruple (a) and *iar3 ill6 cyp94b1 cyp94b3 cyp94c1* pentuple mutant seedlings (b) at 28°C in the absence or presence of 30 µM MeJA. Boxplots show mean as '+' and depict median with Tukey-based whiskers and outliers. Different letters denote significant differences ($p < 0.05$) based on two-way ANOVA with Tukey's HSD. The exact *p*-value for the interaction (genotype × MeJA) is shown at the top. The % decrease is indicated. The number of individually measured seedlings (n) is indicated above the X-axis.

Supplementary References

1 Vu, L. D. *et al.* Temperature-induced changes in the wheat phosphoproteome reveal temperature-regulated interconversion of phosphoforms. *J Exp Bot* **69**, 4609-4624, doi:10.1093/jxb/ery204 (2018).

2 Tian, X. *et al.* Heat shock transcription factor A1b regulates heat tolerance in wheat and Arabidopsis through OPR3 and jasmonate signalling pathway. *Plant Biotechnol J*, doi:10.1111/pbi.13268 (2019).

Supplementary Table

Supplementary Table S1. Primers used for RT-qPCR in this study.

**Primer_ID**	**Sequence**	**Note**
ARP7-Fwd	ACTCTTCCTGATGGACAGGTG	Arabidopsis housekeeping gene
ARP7-Rev	CTCAACGATTCCATGCTCCT	Arabidopsis housekeeping gene
EF1α-Fwd	CTGGAGGTTTTGAGGCTGGTAT	Arabidopsis housekeeping gene
EF1α -Rev	CCAAGGGTGAAAGCAAGAAGA	Arabidopsis housekeeping gene
HSP70-Fwd	GAAGTACAAGGCTGAGGATGAAGAAC	
HSP70-Rev	CTTCTCGTCCTTGATCGTGTTCC	
IAA29-Fwd	TCCTCTGGAATCCGAGTCTTC	
IAA29-Rev	GGTGGCCATCCAACAACTT	
YUC8-Fwd	GACTGCTCGGTTCGATGAGA	
YUC8-Rev	TGAATCACCTCACCGGAAAA	
EXPA8-Fwd	CTCTTTCCGAAGAGTACCATGT	
EXPA8-Rev	GTGTACGTCTCCTGCTCCTC	
EXLA-2-Fwd	TTGTACCAAGGAGGCCAAAC	
EXLA-2-Rev	GGTCACCACGAACCTGAACT	
LOX2-Fwd	AATGAGCCTGTTATCAATGC	
LOX2-Rev	CATACTTAACAACACCAGCTCC	
LOX3-Fwd	CACTGCAATTCACAAGCAACC	
LOX3-Rev	CAAAGGAGGAATCGGAGAAGC	
AOS-Fwd	GCGACGAGAGATCCGAAGA	
AOS-Rev	CTCGCCACCAAAACAACAAA	
OPR3-Fwd	ACGGCGGCACAAGGGAACTCTAAC	
OPR3Rev	GGGAACCATCGGGCAACAAAACTC	
JAR1-Fwd	CTCACTGGTCACCCTGTTCC	
JAR1-Rev	GAAGGCAAAAGCAGTGCGAA	
CYP94B1-Fwd	AAAGAAAAGCCTCGATATCGG	
CYP94B1-Rev	CCATGTGAGCGGTTAGAAGAGG	
CYP94B3-Fwd	TGGCTTACACGAAGGCTTGTC	
CYP94B3-Rev	AGTCCCACGAAACTGGAGGAT	
CYP94C1-Fwd	GGCCCGGATTACGAAGAGTTT	
CYP94C1-Rev	GGCCGGAACTTACCTTCGTT	
IAR3-Fwd	TCATCATCACGCACACCTCTCTTA	
IAR3-Rev	GAAACCCATTTGAAGAAACTCAT	
ILL6-Fwd	GGTCCATGTGTCCCATATCC	
ILL6-Rev	AGCTTCACGGGATACAATGC	
JOX1-Fwd	CGTGGATCACTGTCAATCCT	
JOX1-Rev	CGATGTTCCACGCTCTTGTA	

JOX2-Fwd	CTCATCCCCATGCTTTCATC	
JOX2-Rev	TCCGAGTTCACTATCACTCTATGC	
JOX3-Fwd	GAACCAGCTCCTCATGCTTT	
JOX3-Rev	GGGTTCACGATCACTCTGTG	
JOX4-Fwd	CATGCAGGCTTTAGGAGGAG	
JOX4-Rev	CCTCCAGGGTCAGAATGAGA	
ST2A-Fwd	CTGAGGGCCTACTATATACG	
ST2A-Rev	CGACAAACTTCGGTGTTGAC	
qGUS-Fw	AGACTGTAACCACGCGTCTG	
qGUS-Rv	TCCAGTTGCAACCACCTGTT	
TaActin-Fwd	CTTGTATGCCAGCGGTCGAACA	Wheat housekeeping gene
TaActin-Rev	CTCATAATCAAGGGCCACGTA	Wheat housekeeping gene
TaCDC-Fwd	CAGCTGCTGACTGAGATGGA	Wheat housekeeping gene
TaCDC-Rev	ATGTCTGGCCTGTTGGTAGC	Wheat housekeeping gene
TaJOX-Fwd (~AtJOX1-4)	CTCGACTGGGGCGACTACTA	TraesCS4A02G109200; TraesCS4B02G195000; TraesCS4D02G195700
TaJOX-Rev (~AtJOX1-4)	CCTTGGACAGCACCTTTGAT	
TaJOX-Fwd (~AtJOX1-4)	AGATCCAGGTGCTGACGAAC	TraesCS1A02G070200; TraesCS1B02G088600; TraesCS1D02G072700
TaJOX-Rev (~AtJOX1-4)	CGGATGTAGAGGCGGTACTC	
TaJOX-Fwd (~AtJOX1-4)	ATAACGTCGAGGGGACACAG	TraesCS5A02G154900; TraesCS5B02G153100; TraesCS5D02G159800
TaJOX-Rev (~AtJOX1-4)	CTCGAGGGTTGCAGAAGAAG	
TaJOX-Fwd (~AtJOX1-4)	GTGTACAAGAGCGTGGAGCA	TraesCS3A02G351400; TraesCS3B02G383900; TraesCS3D02G345600
TaJOX-Rev (~AtJOX1-4)	CCCTGTACTCGTCGAAGGTC	
TaST2A-Fwd (~AtST2a)	GCGTTCTTCAGGAATGGGGA	TraesCS2A02G299300; TraesCS2B02G315600; TraesCS2D02G297200
TaST2A-Rev (~AtST2a)	TCGATTCTCTGCGCCATCTC	
TaST2A-Fwd (~AtST2a)	GTGCCCAACAGCGTTTTCTT	TraesCS5A02G347700; TraesCS5D02G353900
TaST2A-Rev (~AtST2a)	GAGCCCCTGAACTTGCTCTC	
TaLOX3-Fwd (~AtLOX3)	ACTTCGACTGCAACTCCTGG	TraesCS4A02G009400; TraesCS4B02G295200; TraesCS4D02G294100
TaLOX3-Rev (~AtLOX3)	ACTTCGACTGCAACTCCTGG	
TaAOS-Fwd (~AtAOS)	TCCTCTTCCACCTCCTCACC	TraesCS5A02G403200; TraesCS5B02G408000; TraesCS5D02G413200
TaLAOS-Rev (~AtAOS)	TCGTCGTTGTAGTTGCCGAA	
TaAOS-Fwd (~AtLAOS)	TACAACCCCAGCGACTCCTT	TraesCS6A02G027900; TraesCS6B02G039500; TraesCS6D02G031100
TaAOS-Rev (~AtAOS)	GAGGAGGTCCATGGCGAAG	
TaOPR3-Fwd (~AtOPR3)	GGCGCTCATGGCTACATCAT	TraesCS7A02G412400; TraesCS7B02G311600; TraesCS7D02G405500
TaOPR3-Rev (~AtOPR3)	ACGGGGTCAGGGGTGTAGAA	
TaOPR3-Fwd (~AtOPR3)	ACTTCGCCAACTACCCCAAG	TraesCS5B02G487600
TaOPR3-Rev (~AtOPR3)	ACGAACCTTGACAGGACGTC	
